# Supplementary material for: Trends and cross-country inequities by region, sex, age in the mortality, incidence, and disability-adjusted life years of COVID-19: Analysis from the Global Burden of Disease Study 2021
Source: PLoS Negl Trop Dis. 2025 Oct 27;19(10):e0013642. doi: 10.1371/journal.pntd.0013642 (PMC12558479; doi:10.1371/journal.pntd.0013642)
Supplement: S1 Fig — The red points are the selected joint points. DALY, Disability-Adjusted Life Year; YLL, Years of Life Lost; AAPC, Average annual percentage change. (DOCX) [file pntd.0013642.s001.docx]

**
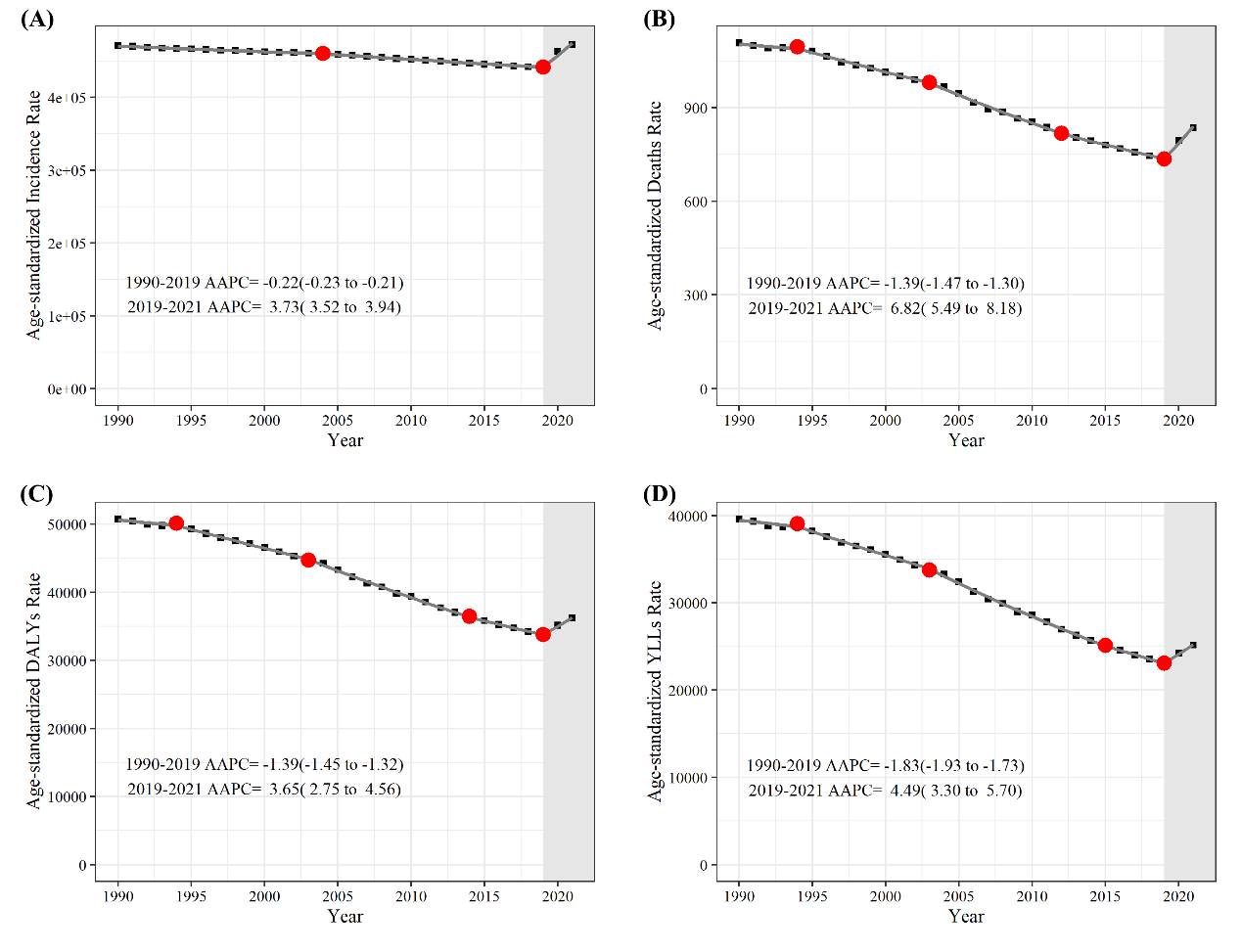
**

**S1 Fig.** **The joinpoint regression analysis on age-standardized rate of COVID-19 incidence, death, DALY, and YLL.**

The red points are the selected joint points. DALY, Disability-Adjusted Life Year; YLL, Years of Life Lost; AAPC, Average annual percentage change.
