# Supplementary material for: Trends and cross-country inequities by region, sex, age in the mortality, incidence, and disability-adjusted life years of COVID-19: Analysis from the Global Burden of Disease Study 2021
Source: PLoS Negl Trop Dis. 2025 Oct 27;19(10):e0013642. doi: 10.1371/journal.pntd.0013642 (PMC12558479; doi:10.1371/journal.pntd.0013642)
Supplement: S2 Fig — The left column is the case number and the right column is the rate. YLL, Years of Life Lost. (DOCX) [file pntd.0013642.s002.docx]

**
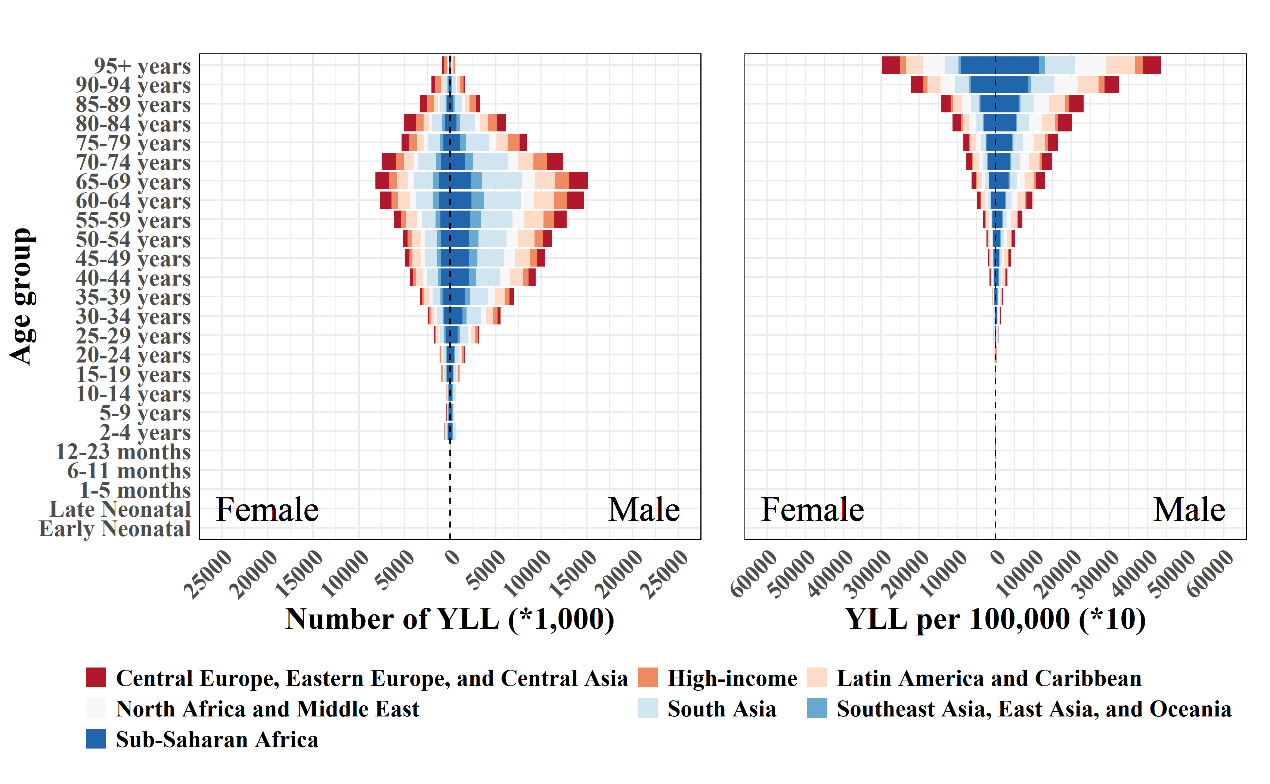
**

**S2 Fig. Age-specific YLL due to COVID-19 by sex and GBD super-region in 2021.**

The left column is the case number and the right column is the rate. YLL, Years of Life Lost.
