# Supplementary material for: Trends and cross-country inequities by region, sex, age in the mortality, incidence, and disability-adjusted life years of COVID-19: Analysis from the Global Burden of Disease Study 2021
Source: PLoS Negl Trop Dis. 2025 Oct 27;19(10):e0013642. doi: 10.1371/journal.pntd.0013642 (PMC12558479; doi:10.1371/journal.pntd.0013642)
Supplement: S3 Fig — The upper panel is the age-standardized rate and the bottom panel is the percentage change in age-standardized rates between 2020 and 2021. YLL, Years of Life Lost. The basemap shapefile was from R package “maps” version 3.4.3. https://cran.rstudio.com/web/packages/maps/index.html. (DOCX) [file pntd.0013642.s003.docx]

**
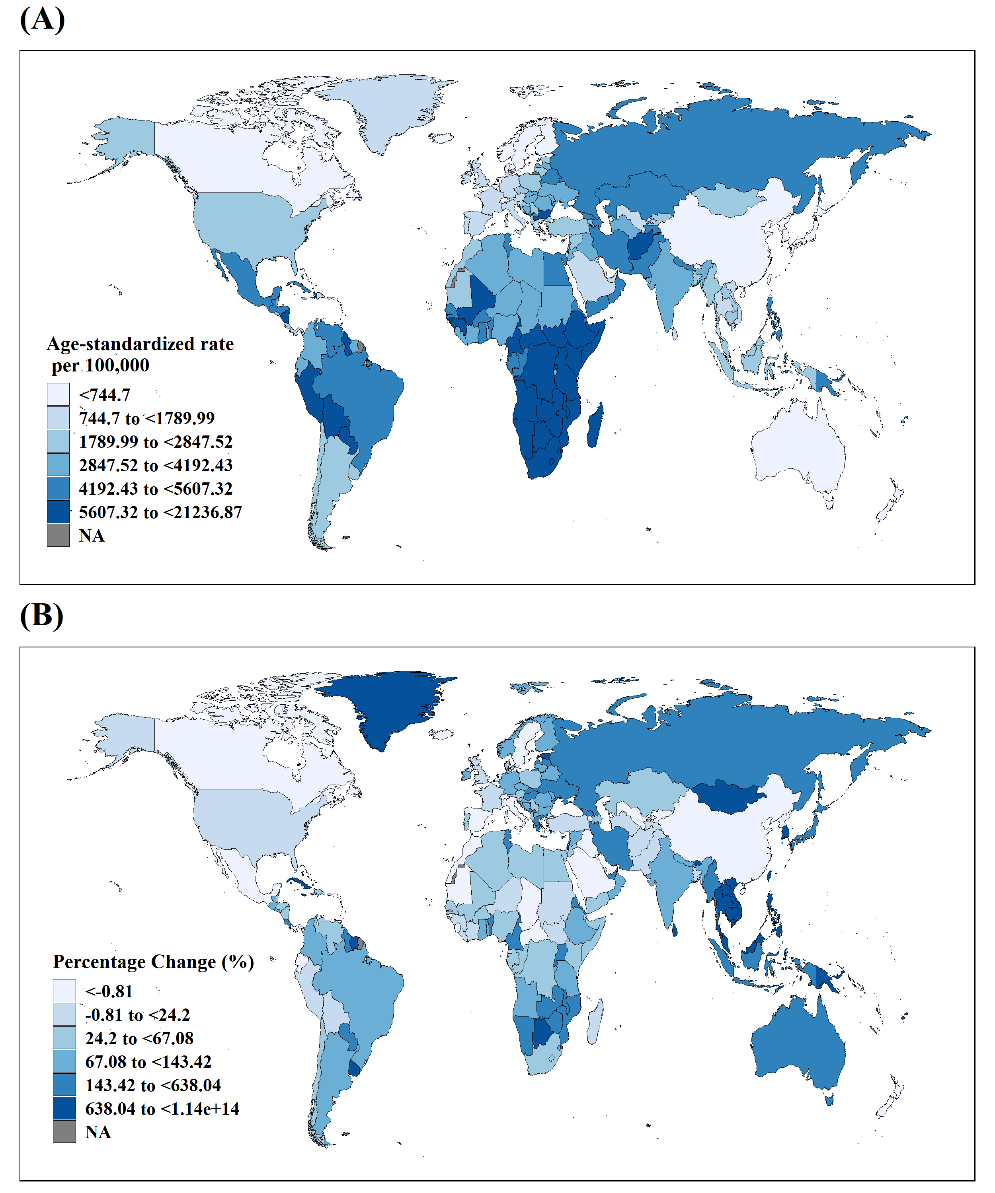
**

**S3 Fig. Country-specific YLL due to COVID-19 in 2021.**

The upper panel is the age-standardized rate and the bottom panel is the percentage change in age-standardized rates between 2020 and 2021. YLL, Years of Life Lost. The basemap shapefile was from R package "maps" version 3.4.3. <https://cran.rstudio.com/web/packages/maps/index.html>.
