# Supplementary material for: Trends and cross-country inequities by region, sex, age in the mortality, incidence, and disability-adjusted life years of COVID-19: Analysis from the Global Burden of Disease Study 2021
Source: PLoS Negl Trop Dis. 2025 Oct 27;19(10):e0013642. doi: 10.1371/journal.pntd.0013642 (PMC12558479; doi:10.1371/journal.pntd.0013642)
Supplement: S4 Fig — The bar graphs represent the inequity indexes and the error bars indicate the corresponding 95% confidence intervals. DALY, Disability-Adjusted Life Year; YLL, Years of Life Lost; SDI, sociodemographic index; EDU15 + , mean educational attainment for those aged 15 and older; TFU25, total fertility rate under the age of 25; LDI, lag-distributed income. (DOCX) [file pntd.0013642.s004.docx]

**
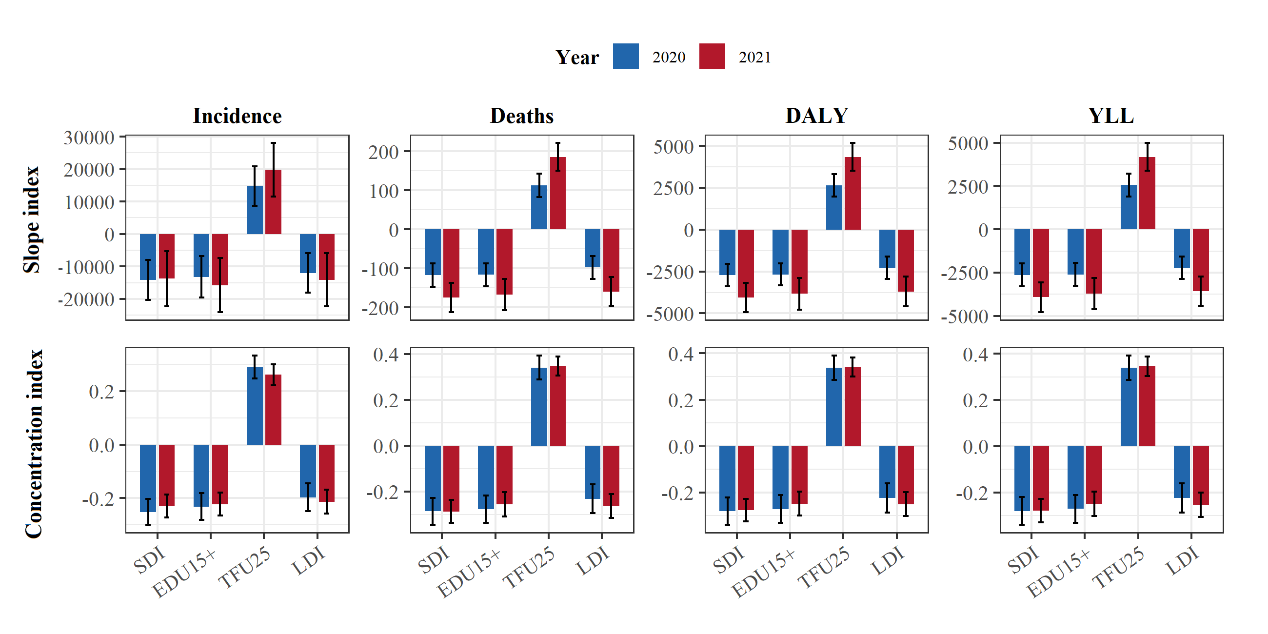
**

**S4 Fig. Inequity indexes related to SDI and its three components for the incidence, death, DALY, and YLL of COVID-19 in 2020 and 2021.**

The bar graphs represent the inequity indexes and the error bars indicate the corresponding 95% confidence intervals. DALY, Disability-Adjusted Life Year; YLL, Years of Life Lost; SDI, sociodemographic index; EDU15+, mean educational attainment for those aged 15 and older; TFU25, total fertility rate under the age of 25; LDI, lag-distributed income.
