# Supplementary material for: Trends and cross-country inequities by region, sex, age in the mortality, incidence, and disability-adjusted life years of COVID-19: Analysis from the Global Burden of Disease Study 2021
Source: PLoS Negl Trop Dis. 2025 Oct 27;19(10):e0013642. doi: 10.1371/journal.pntd.0013642 (PMC12558479; doi:10.1371/journal.pntd.0013642)
Supplement: S5 Fig — YLL, Years of Life Lost; SDI, sociodemographic index. (DOCX) [file pntd.0013642.s005.docx]

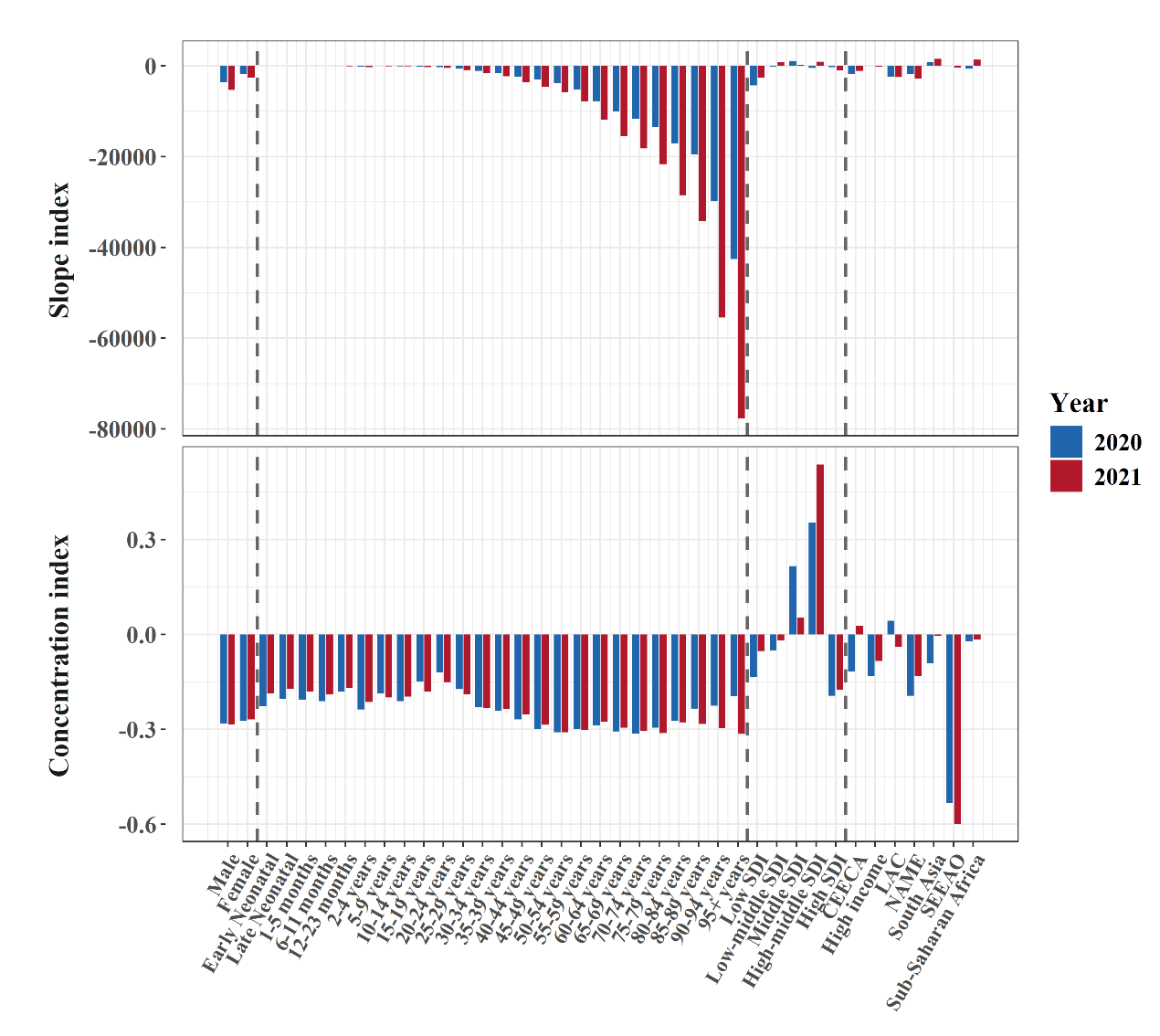


**S5 Fig. Inequity indexes for the YLL of COVID-19 by sex, age groups, SDI, and super-regions in 2020 and 2021.**

YLL, Years of Life Lost; SDI, sociodemographic index.
