# Supplementary material for: Trends and cross-country inequities by region, sex, age in the mortality, incidence, and disability-adjusted life years of COVID-19: Analysis from the Global Burden of Disease Study 2021
Source: PLoS Negl Trop Dis. 2025 Oct 27;19(10):e0013642. doi: 10.1371/journal.pntd.0013642 (PMC12558479; doi:10.1371/journal.pntd.0013642)
Supplement: S6 Fig — SDI, sociodemographic index; CI, confidence interval. Vaccination rate refers to the percentage of total population vaccinated with at least one dose of a COVID-19 vaccine until 31 December 2021. Data was collected from Our World in Data, available at https://ourworldindata.org/coronavirus (Accessed June 25, 2025). (DOCX) [file pntd.0013642.s006.docx]

**
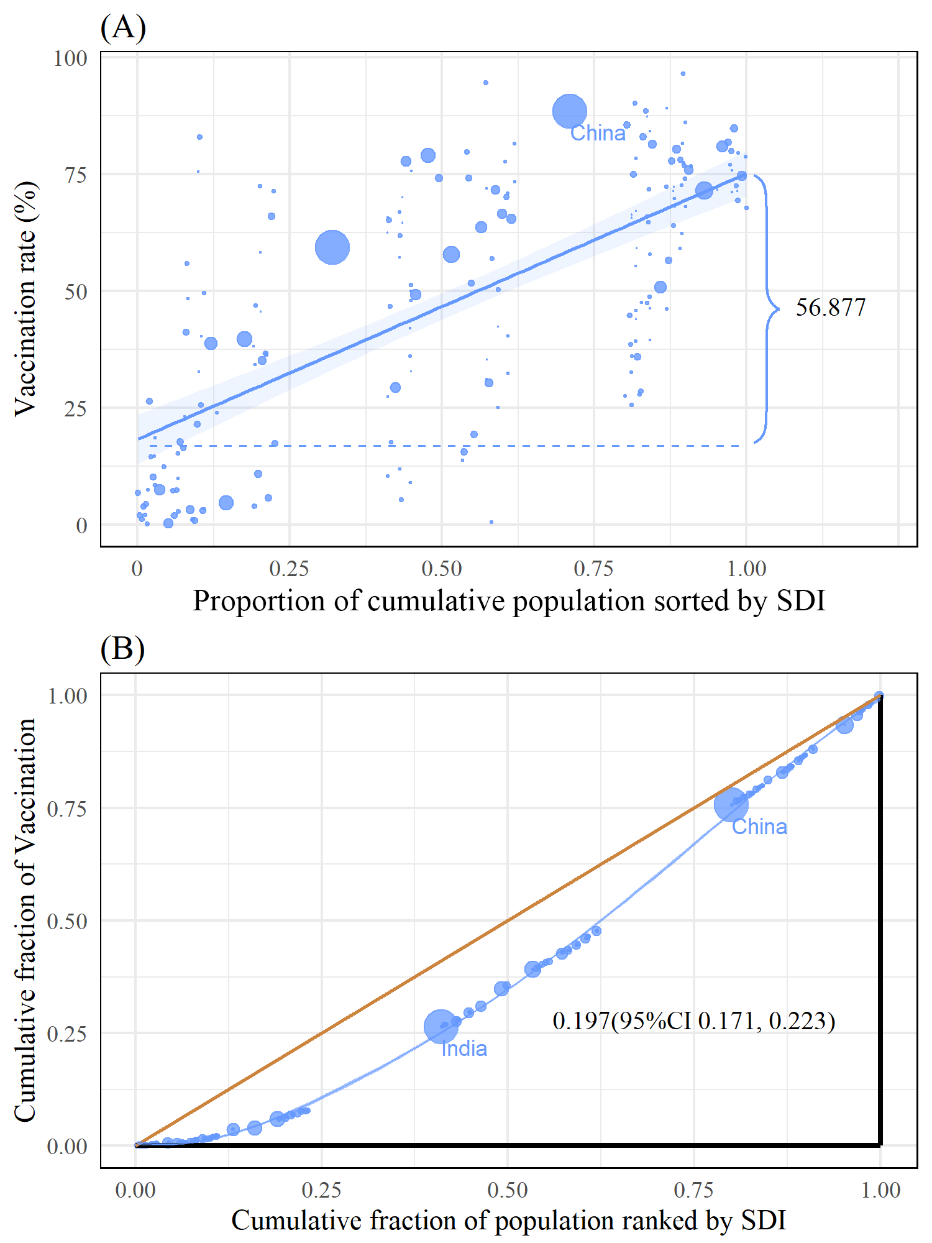
**

**S6 Fig. Inequity regression curve (A) and concentration curve (B) of COVID-19 vaccination rates across the world.**

SDI, sociodemographic index; CI, confidence interval. Vaccination rate refers to the percentage of total population vaccinated with at least one dose of a COVID-19 vaccine until 31 December 2021. Data was collected from Our World in Data, available at <https://ourworldindata.org/coronavirus> (Accessed June 25, 2025).
