# Supplementary material for: Trends and cross-country inequities by region, sex, age in the mortality, incidence, and disability-adjusted life years of COVID-19: Analysis from the Global Burden of Disease Study 2021
Source: PLoS Negl Trop Dis. 2025 Oct 27;19(10):e0013642. doi: 10.1371/journal.pntd.0013642 (PMC12558479; doi:10.1371/journal.pntd.0013642)
Supplement: S1 Table — * Percentage change is the comparison of age-standardized rates for 2020 and 2021. YLL, Years of Life Lost; SDI, sociodemographic index; CI, confidence interval. (DOCX) [file pntd.0013642.s007.docx]

**S1 Table. The case number and age-standardized rates of YLLs for COVID-19 by sex and SDI in 2020 and 2021.**

|  | **2020** | | **2021** | | **Percentage**  **Change in age-standardized rates between 2020 and 2021 (95% CI)** * |
| --- | --- | --- | --- | --- | --- |
|  | **Number (95% CI, *10^6^)** | **Age-standardized rate per 100,000 (95% CI)** | **Number (95% CI, *10^6^)** | **Age-standardized rate per 100,000 (95% CI)** |  |
| Global | 118.40(112.33-126.34) | 1420(1347-1515) | 197.76(187.90-211.51) | 2324(2209-2485) | 63.68(50.54 to 77.97) |
| Sex |  |  |  |  |  |
| Male | 76.61(72.54-81.94) | 1930(1829-2063) | 126.31(119.76-135.39) | 3112(2951 -3332) | 61.20(47.89 to 75.70) |
| Female | 41.79(39.60-44.44) | 958(908-1019) | 71.45(67.51-76.33) | 1606(1517-1716) | 67.72(54.09 to 82.56) |
| SDI |  |  |  |  |  |
| Low SDI | 18.29(17.27-19.69) | 3242(3066-3486) | 30.93(28.96-32.88) | 5361(5028-5686) | 65.36(51.24 to 80.79) |
| Low-middle SDI | 36.98(34.64-39.51) | 2502(2345-2671) | 58.56(55.04-63.31) | 3865(3637-4177) | 54.43(40.36 to 69.91) |
| Middle SDI | 32.05(29.54-35.38) | 1228(1133-1354) | 58.26(53.73-64.94) | 2179(2010-2430) | 77.47(55.57 to 102.46) |
| High-middle SDI | 14.72(13.47-16.15) | 799(731-877) | 29.89(27.34-32.29) | 1591(1456-1718) | 99.10(76.04 to 125.18) |
| High SDI | 16.27(15.67-16.83) | 943(909-976) | 19.90(19.24-20.67) | 1133(1096-1174) | 20.12(14.31 to 26.23) |

* Percentage change is the comparison of age-standardized rates for 2020 and 2021.

YLL, Years of Life Lost; SDI, sociodemographic index; CI, confidence interval.
