# Supplementary material for: Trends and cross-country inequities by region, sex, age in the mortality, incidence, and disability-adjusted life years of COVID-19: Analysis from the Global Burden of Disease Study 2021
Source: PLoS Negl Trop Dis. 2025 Oct 27;19(10):e0013642. doi: 10.1371/journal.pntd.0013642 (PMC12558479; doi:10.1371/journal.pntd.0013642)
Supplement: S2 Table — * Percentage change is the comparison of age-standardized rates for 2020 and 2021. DALY, Disability-Adjusted Life Year; YLL, Years of Life Lost; CI, confidence interval. (DOCX) [file pntd.0013642.s008.docx]

**S2 Table. The case number and age-standardized rates of incidence, deaths, DALYs, and YLLs for COVID-19 by age and region in 2020 and 2021.**

|  | **2020** | | **2021** | | **Percentage**  **Change in age-standardized rates between 2020 and 2021 (95% CI)** * |
| --- | --- | --- | --- | --- | --- |
|  | **Number (95% CI)** | **Age-standardized rate per 100,000 (95% CI)** | **Number (95% CI)** | **Age-** **standardized rate per 100,000 (95% CI)** |  |
| **Incidence** |  |  |  |  |  |
| Age group |  |  |  |  |  |
| Early Neonatal | 3149.39  (2990.9-3284.1) | 126.62  (120.25-132.04) | 4254.92  (4054.93-4460.56) | 173.58  (165.43-181.97) | 37.09  (28.25 to 46.54) |
| Late Neonatal | 28046.02  (26636.73-29245.48) | 378.87  (359.83-395.08) | 37915.82  (36139.68-39743.03) | 519.82  (495.47-544.87) | 37.2  (28.38 to 46.64) |
| 1-5 months | 1521480.06  (1445869.94-1586206.4) | 2782.79  (2644.49-2901.17) | 2058132.18  (1961927.39-2155602.88) | 3828.58  (3649.62-4009.9) | 37.58  (28.81 to 46.95) |
| 6-11 months | 2090875.34  (1987393.18-2179034.32) | 3239.43  (3079.1-3376.01) | 2831051.2  (2698792.63-2964574.62) | 4480.06  (4270.76-4691.36) | 38.3  (29.52 to 47.67) |
| 12-23 months | 8405552.56  (7985003.28-8761943.72) | 6395.03  (6075.07-6666.18) | 11392075.32  (10851053.81-11916843.9) | 8871.59  (8450.27-9280.25) | 38.73  (29.9 to 48.16) |
| 2-4 years | 75967607.11  (72210687.48-79224269.75) | 18562.87  (17644.85-19358.64) | 103265619.35  (98400545.42-107961864.73) | 25620  (24412.99-26785.13) | 38.02  (29.28 to 47.34) |
| 5-9 years | 164789759.68  (157220015.44-172142462.52) | 24201.29  (23089.58-25281.12) | 230330094.41  (219427295.97-240470914.71) | 33524.33  (31937.44-35000.32) | 38.52  (29.89 to 47.73) |
| 10-14 years | 165697319.93  (158050313.56-173164996.35) | 25085.89  (23928.17-26216.47) | 233560538.32  (222022978.86-244150485.4) | 35035.69  (33304.98-36624.26) | 39.66  (30.77 to 49.16) |
| 15-19 years | 161477332.48  (154120180.24-168755509.27) | 26163.63  (24971.57-27342.88) | 228112165.52  (216914751.78-238602177.81) | 36557.62  (34763.1-38238.76) | 39.73  (30.84 to 49.21) |
| 20-24 years | 153584267.9  (146695631.17-160516426.47) | 25776.12  (24620-26939.55) | 217042181.96  (206914473.67-226783537.89) | 36345.85  (34649.86-37977.13) | 41.01  (32.24 to 50.35) |
| 25-29 years | 142746221.65  (136479758.04-148977246.25) | 24151.22  (23090.99-25205.44) | 201234058.58  (192255026.07-210253169.85) | 34203.51  (32677.36-35736.48) | 41.62  (33.03 to 50.77) |
| 30-34 years | 131307500.16  (125726323.7-136841998.97) | 21738.39  (20814.41-22654.65) | 184687049.01  (176986949.14-192348816.93) | 30553.04  (29279.2-31820.53) | 40.55  (32.45 to 49.14) |
| 35-39 years | 116708779.32  (111692705.23-121494855.92) | 21294.17  (20378.96-22167.42) | 163782213.56  (156875078.4-170360881.21) | 29201.66  (27970.15-30374.61) | 37.13  (29.3 to 45.44) |
| 40-44 years | 100486094.39  (96114435.24-104636143.26) | 20333.41  (19448.8-21173.17) | 140401327.21  (134369186.8-146099412.45) | 28066.19  (26860.36-29205.23) | 38.03  (30.05 to 46.5) |
| 45-49 years | 88461748.42  (84669197.24-92103333.11) | 18698.9  (17897.23-19468.65) | 123371532.28  (118098156.25-128421421.3) | 26054.98  (24941.29-27121.47) | 39.34  (31.32 to 47.85) |
| 50-54 years | 78898950.04  (75404915.08-82083483.81) | 17945.71  (17150.98-18670.03) | 110075940.18  (105706233.7-114240798.8) | 24740.45  (23758.32-25676.53) | 37.86  (30.17 to 46.01) |
| 55-59 years | 70901113.74  (67552922.06-73762951.77) | 18458.64  (17586.96-19203.7) | 99576182.11  (95655482.87-103208857.04) | 25162.78  (24172.03-26080.76) | 36.32  (28.65 to 44.45) |
| 60-64 years | 58940867.94  (56102777.86-61289750.42) | 18685.19  (17785.47-19429.82) | 83195309.4  (79753795.69-86289687.84) | 25994.65  (24919.33-26961.5) | 39.12  (31.15 to 47.57) |
| 65-69 years | 42483280.02  (40505195.04-44166436.59) | 15760.39  (15026.56-16384.8) | 59902744.14  (57442995.37-62123493.53) | 21716.31  (20824.59-22521.39) | 37.79  (30 to 46.04) |
| 70-74 years | 27526552.75  (26370481.44-28614847.66) | 14040.85  (13451.16-14595.97) | 38513353.74  (37012185.58-39889970.58) | 18710.41  (17981.11-19379.19) | 33.26  (26.09 to 40.83) |
| 75-79 years | 16316567.27  (15677532.97-16957797.4) | 12613.26  (12119.27-13108.96) | 22523081.44  (21672710.24-23298303.71) | 17077.9  (16433.11-17665.7) | 35.4  (28.37 to 42.81) |
| 80-84 years | 10110352.72  (9748489.68-10496178.57) | 11723.09  (11303.51-12170.46) | 14151586.08  (13624199.24-14631709.98) | 16157.9  (15555.75-16706.1) | 37.83  (30.93 to 45.09) |
| 85-89 years | 4726679.47  (4566960.99-4896735.28) | 10599.61  (10241.44-10980.96) | 6489643.9  (6283132.56-6692699.89) | 14193.77  (13742.1-14637.88) | 33.91  (27.76 to 40.36) |
| 90-94 years | 1798946.86  (1740447.26-1867102.87) | 10314.29  (9978.88-10705.06) | 2445494.36  (2370415.9-2522651.71) | 13670.09  (13250.41-14101.4) | 32.54  (26.45 to 38.91) |
| ≥95 years | 552012.46  (532943.59-573911.43) | 10558.73  (10193.99-10977.61) | 734222.58  (712396.26-756170) | 13471.21  (13070.75-13873.89) | 27.58  (21.65 to 33.8) |
| Region |  |  |  |  |  |
| Central Europe, eastern Europe, and central Asia | 133826762.47  (122468466.13-145577335.16) | 31074.9  (28371.42-33887.24) | 219279907.84  (201318987.77-237403753.89) | 50864.33  (46838.47-55109.11) | 63.68  (45.12 to 84.62) |
| Central Asia | 29415489.15  (21256527.84-35270788.02) | 31060.61  (22496.94-37232.58) | 41954381.42  (31353051.48-52702044.69) | 43721.56  (32721.73-54890.7) | 40.76  (-0.53 to 99.19) |
| Central Europe | 33643666.3  (30460506.7-36627101.85) | 28121.53  (25462.09-30612.69) | 51821889.05  (46727372.27-56311875.16) | 43678.28  (39325.5-47435.3) | 55.32  (36.33 to 76.95) |
| Eastern Europe | 70767607.03  (62593079.76-78722591.87) | 32795.59  (29017.19-36410.37) | 125503637.37  (111687136.48-138889764.27) | 58681.12  (52343.53-64812.49) | 78.93  (53.25 to 108.91) |
| High income | 115412215.05  (110884210.69-121248289.5) | 10995.35  (10548.96-11558.85) | 149039171.89  (143673321.29-155361840.76) | 14180.15  (13654.9-14795.17) | 28.97  (21.33 to 37.08) |
| Australasia | 83067.75  (71217.96-97945) | 272.71  (233.91-321.53) | 340300.17  (307196.94-373550.69) | 1102.76  (996.05-1210.14) | 304.38  (235.18 to 387.87) |
| High-income Asia Pacific | 2257938.95  (2115353.81-2403666.68) | 1227.93  (1149.51-1311.36) | 6376892.45  (5869068.95-7464219.96) | 3543.21  (3250.39-4181.21) | 188.55  (149.11 to 234.23) |
| High-income North America | 58163849.33  (55846860.64-60669990.88) | 16084.73  (15442.83-16783.58) | 66211214.39  (62958009.43-69532122.04) | 18279.18  (17380.29-19191.74) | 13.64  (6.52 to 21.24) |
| Southern Latin America | 7101294.68  (5438622.17-9835754.24) | 10520.03  (8044.8-14592.99) | 10889559.92  (8798054.91-14577764.75) | 16051.75  (12960.48-21515.9) | 52.58  (1.29 to 129.85) |
| Western Europe | 47806064.35  (44641948.96-52501449.03) | 11219.34  (10465.14-12343.11) | 65221204.96  (61856449.41-69516456.81) | 15361.7  (14572.67-16375.27) | 36.92  (23.62 to 51.66) |
| Latin America and Caribbean | 182386505.07  (173855522.46-190588857.81) | 30584.31  (29159.44-31946.87) | 197104662.79  (185553028.94-207700214.9) | 32804.74  (30882.96-34566.92) | 7.26  (-0.22 to 15.3) |
| Andean Latin America | 28114408.4  (23451323.5-32873258.24) | 42937.57  (35808.64-50212.3) | 24340143.71  (19634782.84-29412648.35) | 36607.03  (29528.52-44235.95) | -14.74  (-34.37 to 10.76) |
| Caribbean | 4632543.2  (2744595.39-6406482.02) | 9856.45  (5817.22-13647.63) | 9415078.14  (6135437.94-13670217.8) | 19743.14  (12753.23-28910.43) | 100.31  (13.25 to 254.29) |
| Central Latin America | 83302455.1  (78522862.51-87876464.31) | 32644.05  (30772-34434.86) | 80481509.38  (74046494.33-86344679.36) | 31406.37  (28888.95-33698.09) | -3.79  (-12.5 to 5.79) |
| Tropical Latin America | 66337098.38  (60749967.85-71847601.17) | 29031.3  (26575.57-31451.62) | 82867931.56  (74935767.77-91531477.8) | 35921.59  (32493.42-39681.94) | 23.73  (8.58 to 41) |
| North Africa and Middle East | 194510244.01  (153487760.63-225159821.56) | 32223.37  (25486.79-37343.53) | 208665193.49  (169750343.58-241914598.25) | 34139.9  (27857.95-39516.27) | 5.95  (-17.57 to 36.18) |
| South Asia | 559344199.18  (510887576.87-616891557.66) | 29969.18  (27388.56-33044.65) | 787565590.59  (708229013.92-866657343.95) | 41742.86  (37545.96-45919.03) | 39.29  (21.37 to 59.85) |
| Southeast Asia, east Asia, and Oceania | 65583885.14  (59902003.71-70845978.44) | 3333.69  (3046.1-3599.99) | 222354547.11  (202218106.33-242690959.78) | 11291.61  (10271.44-12322.55) | 238.71  (199.49 to 283.08) |
| East Asia | 2433080.79  (2120902.29-2817047.23) | 177.54  (154.81-205.85) | 196997.92  (165434.3-245032.96) | 14.43  (12.13-17.85) | -91.87  (-93.64 to -89.62) |
| Oceania | 177053.3  (107226.9-305925.16) | 1283.35  (782.08-2180.84) | 4808370.7  (2496465.53-7157053.68) | 32963.52  (17227.58-48996.67) | 2468.56  (1140.96 to 5216.45) |
| Southeast Asia | 62973751.05  (57320020.01-68424832.39) | 9060.5  (8249.7-9843.72) | 217349178.49  (197481814.11-237502307.66) | 31065.66  (28230.33-33925.73) | 242.87  (201.96 to 289.32) |
| Sub-Saharan Africa | 374467246.74  (350814916.13-391857972.93) | 35248.55  (33022.03-36880.06) | 495708693.83  (465749450.56-524556977.35) | 45417.11  (42698.67-48008.69) | 28.85  (18.93 to 39.59) |
| Central Sub-Saharan Africa | 59396956.07  (39563899.08-69277956.21) | 46494.85  (30970.95-54215.83) | 58278702.81  (43651392.01-71094692.7) | 44202.05  (33151.7-53933.33) | -4.93  (-32.55 to 33.99) |
| Eastern Sub-Saharan Africa | 110094137.74  (103755399.71-115908427.75) | 27611.58  (26055.45-29046.12) | 232976193.39  (216027744.62-247769055.57) | 56618.8  (52586.97-60141.97) | 105.05  (88.17 to 123.45) |
| Southern Sub-Saharan Africa | 23012006.95  (20765935.57-25111093.66) | 28776.62  (25975.96-31397.7) | 38692437.1  (32371314.4-42252846.41) | 47661.07  (40023.01-52003.06) | 65.62  (41.55 to 93.79) |
| Western Sub-Saharan Africa | 181964145.98  (170595249.23-193566140.62) | 40094.05  (37650.04-42636.9) | 165761360.53  (145444226.6-185807419.18) | 35642.69  (31251.9-39893.91) | -11.1  (-22.43 to 1.87) |
| **Deaths** |  |  |  |  |  |
| Age group |  |  |  |  |  |
| Early Neonatal | 0.34  (0.32-0.37) | 0.01  (0.01-0.01) | 0.59  (0.55-0.64) | 0.02  (0.02-0.03) | 75.32  (57.36 to 95.34) |
| Late Neonatal | 3.14  (2.92-3.42) | 0.04  (0.04-0.05) | 5.37  (4.98-5.88) | 0.07  (0.07-0.08) | 73.81  (54.78 to 95.19) |
| 1-5 months | 169.54  (157.63-183.54) | 0.31  (0.29-0.34) | 286.6  (264.95-314.62) | 0.53  (0.49-0.59) | 71.93  (53.17 to 92.99) |
| 6-11 months | 234.06  (216.64-256.01) | 0.36  (0.34-0.4) | 394.14  (363.32-430.81) | 0.62  (0.57-0.68) | 72  (52.55 to 93.93) |
| 12-23 months | 997.57  (929.09-1069.1) | 0.76  (0.71-0.81) | 1643.97  (1528.03-1783.59) | 1.28  (1.19-1.39) | 68.68  (51.91 to 87.31) |
| 2-4 years | 8499.51  (7968.28-9180.21) | 2.08  (1.95-2.24) | 14384.54  (13377.36-15684.28) | 3.57  (3.32-3.89) | 71.83  (54.35 to 91.3) |
| 5-9 years | 7050.82  (5762.8-8105.97) | 1.04  (0.85-1.19) | 11392.28  (8753.83-13573.85) | 1.66  (1.27-1.98) | 60.13  (22.36 to 109.56) |
| 10-14 years | 8552.86  (6881.36-9827.15) | 1.29  (1.04-1.49) | 14404.82  (10682.07-16836.25) | 2.16  (1.6-2.53) | 66.88  (26.83 to 119.56) |
| 15-19 years | 17031.2  (16197.37-18124.41) | 2.76  (2.62-2.94) | 26849.74  (25322.14-28537.81) | 4.3  (4.06-4.57) | 55.93  (43.6 to 69.32) |
| 20-24 years | 25526.36  (24325.95-27030.17) | 4.28  (4.08-4.54) | 40738.72  (38452.3-43287.91) | 6.82  (6.44-7.25) | 59.24  (47.07 to 72.43) |
| 25-29 years | 47855.01  (45512.08-50883.79) | 8.1  (7.7-8.61) | 78490.17  (74275.05-83566.93) | 13.34  (12.62-14.2) | 64.77  (51.86 to 78.78) |
| 30-34 years | 81228.46  (77165.8-86803.31) | 13.45  (12.78-14.37) | 137969.11  (130867.59-147235.4) | 22.82  (21.65-24.36) | 69.73  (56.07 to 84.58) |
| 35-39 years | 112222.6  (106591.9-119830.2) | 20.48  (19.45-21.86) | 195367.16  (185514.22-208751.33) | 34.83  (33.08-37.22) | 70.12  (56.45 to 84.98) |
| 40-44 years | 165329.88  (156746.16-176604.72) | 33.45  (31.72-35.74) | 287079.32  (272441.04-307953.57) | 57.39  (54.46-61.56) | 71.54  (57.37 to 86.98) |
| 45-49 years | 207928.35  (196523.67-222732.16) | 43.95  (41.54-47.08) | 355363.20  (336798.33-382259.9) | 75.05  (71.13-80.73) | 70.76  (56.09 to 86.8) |
| 50-54 years | 253474.45  (239533.76-271867.31) | 57.65  (54.48-61.84) | 426752.99  (403680.17-459681.47) | 95.92  (90.73-103.32) | 66.37  (51.82 to 82.31) |
| 55-59 years | 336139.62  (317250.65-360168.41) | 87.51  (82.59-93.77) | 564466.97  (533981.27-608514.58) | 142.64  (134.94-153.77) | 63.00  (48.69 to 78.67) |
| 60-64 years | 460742.96  (435414.63-492118.54) | 146.06  (138.03-156.01) | 774823.92  (733355.9-833537.42) | 242.10  (229.14-260.44) | 65.75  (51.6 to 81.22) |
| 65-69 years | 564336.73  (534369.15-604090.89) | 209.36  (198.24-224.1) | 957490.05  (908927.89-1026645.24) | 347.12  (329.51-372.19) | 65.80  (51.96 to 80.9) |
| 70-74 years | 585514.47  (556122.76-623656.83) | 298.66  (283.67-318.12) | 989819.71  (942492.97-1053039.88) | 480.87  (457.88-511.58) | 61.01  (48.59 to 74.47) |
| 75-79 years | 539481.75  (513974.3-573392.16) | 417.04  (397.32-443.25) | 861732.26  (821102.63-915109.63) | 653.4  (622.59-693.87) | 56.68  (44.99 to 69.3) |
| 80-84 years | 550980.8  (526142.15-583216.87) | 638.87  (610.07-676.25) | 888750.86  (849246.38-939020.94) | 1014.75  (969.65-1072.15) | 58.84  (47.75 to 70.75) |
| 85-89 years | 427744.07  (408943.22-451334.38) | 959.22  (917.06-1012.12) | 658823.18  (629692.27-693728.55) | 1440.94  (1377.23-1517.28) | 50.22  (40.15 to 61.02) |
| 90-94 years | 280590.62  (268424.68-294515.06) | 1608.77  (1539.02-1688.61) | 426150.76  (408299.65-448326.27) | 2382.14  (2282.36-2506.1) | 48.07  (38.6 to 58.19) |
| ≥95 years | 120167.24  (114872.4-125730.03) | 2298.52  (2197.24-2404.93) | 174373.54  (166599.29-183808.6) | 3199.33  (3056.69-3372.44) | 39.19  (30.18 to 48.82) |
| Region |  |  |  |  |  |
| Central Europe, eastern Europe, and central Asia | 467410.85  (412199.1-524327.14) | 72.93  (64.28-81.84) | 1096987.17  (977902.86-1205348.85) | 168.82  (150.16-185.76) | 131.48  (97.25 to 171.66) |
| Central Asia | 87300.03  (64322.19-106717.49) | 122.71  (91.13-149.57) | 114977.94  (86202.31-138034.03) | 157.29  (118.34-188.56) | 28.18  (-7.52 to 77.64) |
| Central Europe | 162410.48  (127078.93-197765.29) | 72.07  (56.22-87.83) | 307027.86  (240919.72-370282.51) | 136.32  (106.88-164.5) | 89.13  (39.48 to 156.47) |
| Eastern Europe | 217700.35  (188023.75-252343.66) | 62.83  (54.1-72.95) | 674981.37  (588261.64-762300.35) | 191.70  (166.38-217.33) | 205.08  (149.68 to 272.78) |
| High income | 930321.16  (907058.99-951493.47) | 41.81  (40.79-42.77) | 1074180.09  (1057485.88-1091658.52) | 48.07  (47.33-48.88) | 14.96  (11.71 to 18.3) |
| Australasia | 977.07  (931.05-1017.92) | 1.69  (1.61-1.77) | 3444.39  (2795.93-4032.79) | 5.95  (4.83-6.97) | 251.56  (192.12 to 323.09) |
| High-income Asia Pacific | 4785.42  (4073.66-5777.85) | 0.88  (0.75-1.08) | 26230.74  (23845.46-28997.56) | 4.88  (4.46-5.34) | 452.43  (348.24 to 580.85) |
| High-income North America | 456200.37  (440864.7-472017.92) | 72.77  (70.3-75.32) | 509132.82  (499381.17-520092.45) | 80.46  (78.91-82.17) | 10.57  (6.23 to 15.08) |
| Southern Latin America | 64656.28  (62706.71-66679.82) | 74.1  (71.87-76.45) | 107800.42  (106701.21-108895.24) | 121.39  (120.14-122.63) | 63.83  (58.58 to 69.24) |
| Western Europe | 403702.02  (390231.4-416266.19) | 38.98  (37.66-40.19) | 427571.73  (416616.89-439300.98) | 41.65  (40.52-42.86) | 6.83  (2.35 to 11.51) |
| Latin America and Caribbean | 799445.16  (731242.69-866358.56) | 133.66  (122.3-144.84) | 1197043.42  (1115135.76-1298487.86) | 195.41  (182.19-211.99) | 46.2  (30.49 to 63.8) |
| Andean Latin America | 163847.17  (141548.34-190298.11) | 284.67  (246.28-330.39) | 183711.24  (173714.98-192201.12) | 317.33  (300.15-331.91) | 11.48  (-4.62 to 30.29) |
| Caribbean | 20107.5  (11175.11-32342.49) | 37.97  (21.05-61.22) | 86528.08  (64852.31-115143.03) | 158.96  (118.74-212.26) | 318.61  (128.54 to 666.76) |
| Central Latin America | 413552.51  (356001.06-472864.23) | 171.01  (147.3-195.36) | 488994.44  (418544.53-570060.29) | 198.53  (170.26-231.2) | 16.09  (-5.72 to 42.94) |
| Tropical Latin America | 201937.98  (187312.35-219463.39) | 82.47  (76.49-89.59) | 437809.65  (409574.01-466380.28) | 171.86  (160.77-183.11) | 108.4  (88.07 to 130.92) |
| North Africa and Middle East | 482828.64  (415807.09-537748.2) | 123.9  (107.1-137.35) | 698057.42  (613336.69-773665.81) | 172.44  (151.89-190.78) | 39.17  (17.86 to 64.33) |
| South Asia | 1315964.12  (1230782.83-1401479.27) | 101.81  (95.23-108.49) | 2061534.06  (1985879.5-2156573.06) | 156.52  (150.87-163.7) | 53.73  (42.35 to 66.03) |
| Southeast Asia, east Asia, and Oceania | 147051.94  (57221.25-312730.59) | 5.71  (2.22-12.23) | 605539.32  (423017.05-953914.62) | 23.2  (16.25-36.45) | 306.44  (52.74 to 981.55) |
| East Asia | 27522.95  (5985.23-139892.64) | 1.46  (0.31-7.31) | 3031.86  (692.91-10397.29) | 0.19  (0.04-0.65) | -86.87  (-99.26 to 133.69) |
| Oceania | 1129.3  (327.26-2327.34) | 18.31  (5.77-37.23) | 10765.44  (2972.7-20899.18) | 165.61  (47.09-320.15) | 804.44  (174.99 to 2874.64) |
| Southeast Asia | 118399.7  (47604.66-227719.48) | 19.68  (7.8-38.06) | 591742.01  (417699.97-928412.99) | 99.71  (70.64-156.15) | 406.76  (110.11 to 1122.27) |
| Sub-Saharan Africa | 658780.53  (615260.65-705900.35) | 158.92  (148.77-170.02) | 1154212.47  (1069740.71-1238006.91) | 271.03  (251.67-290.2) | 70.54  (54.68 to 88.01) |
| Central Sub-Saharan Africa | 75896.83  (68291.13-84364.07) | 165.9  (150.03-184.06) | 124870.37  (108417.71-147243.59) | 266.45  (231.29-314.57) | 60.61  (33.23 to 93.62) |
| Eastern Sub-Saharan Africa | 230250.78  (213611.33-249303.15) | 155.13  (144.06-167.91) | 477545.34  (429049.41-514926.65) | 315.2  (283-339.66) | 103.18  (80.52 to 128.69) |
| Southern Sub-Saharan Africa | 128674.08  (126595.61-130634.37) | 254.46  (250.74-258) | 217754.29  (205355.56-227210.46) | 420.68  (398.71-437.35) | 65.32  (57.56 to 73.47) |
| Western Sub-Saharan Africa | 223958.85  (203037.64-243864.02) | 130.47  (118.33-142.14) | 334042.47  (301457.02-360357.16) | 190.26  (171.53-205.43) | 45.83  (28.37 to 65.67) |
| **DALY** |  |  |  |  |  |
| Age group |  |  |  |  |  |
| Early Neonatal | 35.86  (31.66-45.15) | 1.44  (1.27-1.82) | 66.64  (56.33-89.73) | 2.72  (2.3-3.66) | 88.56  (37.84 to 157.94) |
| Late Neonatal | 328.9  (288.89-411.49) | 4.44  (3.9-5.56) | 605.71  (512.34-810.71) | 8.3  (7.02-11.11) | 86.91  (37.24 to 154.54) |
| 1-5 months | 17744.8  (15587.46-21885.29) | 32.46  (28.51-40.03) | 32339.27  (27346.83-43745) | 60.16  (50.87-81.38) | 85.36  (36.02 to 152.59) |
| 6-11 months | 24375.32  (21259.01-30016.46) | 37.77  (32.94-46.5) | 44286.27  (37205.26-59592.55) | 70.08  (58.88-94.3) | 85.57  (36.1 to 153.04) |
| 12-23 months | 102260.33  (90963.28-125887.08) | 77.8  (69.21-95.78) | 181811.84  (154484.39-246678.5) | 141.59  (120.3-192.1) | 81.99  (34.05 to 147.06) |
| 2-4 years | 860579.2  (760761.98-1075440.9) | 210.28  (185.89-262.79) | 1573015.01  (1328728.69-2125121.14) | 390.26  (329.66-527.24) | 85.59  (35.81 to 153.61) |
| 5-9 years | 845939.43  (623964.43-1270502.62) | 124.24  (91.64-186.59) | 1641943.46  (1086871.16-2863998.31) | 238.98  (158.19-416.85) | 92.36  (-0.82 to 273.11) |
| 10-14 years | 925721.45  (682156.99-1361640.46) | 140.15  (103.28-206.15) | 1827424.08  (1225688.57-3070995.4) | 274.13  (183.86-460.67) | 95.59  (4.78 to 265.12) |
| 15-19 years | 1493833.37  (1303471.02-1946470.13) | 242.04  (211.2-315.38) | 2655765.13  (2173446.84-3901158.81) | 425.62  (348.32-625.21) | 75.85  (19.05 to 159.73) |
| 20-24 years | 2293337.81  (1887835.76-3162694.47) | 384.89  (316.84-530.8) | 4362356.4  (3227989.49-6834450.74) | 730.52  (540.56-1144.5) | 89.8  (15.34 to 212.34) |
| 25-29 years | 3532876.48  (3123698.72-4350521.4) | 597.73  (528.5-736.06) | 6436593.3  (5336772.25-8706876.9) | 1094.02  (907.08-1479.9) | 83.03  (33.69 to 150.58) |
| 30-34 years | 5186914.59  (4768488.55-5936297.36) | 858.71  (789.44-982.77) | 9391361.45  (8314106.55-11505562.7) | 1553.63  (1375.41-1903.38) | 80.93  (47.56 to 121.83) |
| 35-39 years | 6373107.44  (5948539.19-7059976.28) | 1162.81  (1085.34-1288.13) | 11615542.09  (10561189.26-13516506.85) | 2071.00  (1883.01-2409.93) | 78.10  (52.65 to 107.8) |
| 40-44 years | 8303926.74  (7810963.88-8957329.35) | 1680.3  (1580.55-1812.52) | 14883981.26  (13835925.11-16571251.49) | 2975.3  (2765.8-3312.59) | 77.07  (57.85 to 98.63) |
| 45-49 years | 9282586.06  (8732448.9-9992446.51) | 1962.14  (1845.85-2112.19) | 16284545.14  (15239779.18-17956668.04) | 3439.15  (3218.51-3792.29) | 75.28  (57.40 to 95.18) |
| 50-54 years | 9985143.91  (9401508.85-10751780.35) | 2271.14  (2138.39-2445.51) | 17188088.74  (16134247.94-18754296.29) | 3863.16  (3626.3-4215.18) | 70.1  (53.62 to 88.34) |
| 55-59 years | 11533799.58  (10879775.68-12357609.78) | 3002.75  (2832.48-3217.22) | 19711619.50  (18586749.3-21425269.4) | 4981.10  (4696.85-5414.14) | 65.88  (50.64 to 82.67) |
| 60-64 years | 13540894.83  (12800025.18-14480287.82) | 4292.68  (4057.81-4590.48) | 23078663.87  (21858287-24907009.41) | 7211.00  (6829.69-7782.28) | 67.98  (53.43 to 83.92) |
| 65-69 years | 13902538.49  (13159560.53-14879811.59) | 5157.54  (4881.91-5520.09) | 23835441.82  (22626825.97-25527165.07) | 8640.97  (8202.82-9254.27) | 67.54  (53.62 to 82.73) |
| 70-74 years | 11846096.16  (11267623.85-12600627.17) | 6042.50  (5747.43-6427.38) | 20205999.51  (19272157.36-21494978.67) | 9816.40  (9362.72-10442.61) | 62.46  (50.16 to 75.75) |
| 75-79 years | 8695197.62  (8292827.95-9244233.13) | 6721.68  (6410.64-7146.11) | 14026034.99  (13374108.61-14896544.88) | 10635.10  (10140.78-11295.15) | 58.22  (46.49 to 70.9) |
| 80-84 years | 6937425.83  (6625282.05-7351541.62) | 8044.04  (7682.11-8524.21) | 11279325.29  (10770191.71-11926431.44) | 12878.43  (12297.12-13617.28) | 60.10  (48.79 to 72.27) |
| 85-89 years | 4261302.67  (4072069.69-4510499.04) | 9556.00  (9131.64-10114.82) | 6620263.54  (6328782.5-6982869.93) | 14479.45  (13841.94-15272.52) | 51.52  (41.09 to 62.72) |
| 90-94 years | 2428080.82  (2324275.53-2550534.55) | 13921.44  (13326.27-14623.53) | 3705804.45  (3551830.59-3896468.42) | 20715.12  (19854.42-21780.91) | 48.8  (39.32 to 58.92) |
| ≥95 years | 978714.32  (935760.34-1023249.54) | 18720.55  (17898.94-19572.41) | 1426717.18  (1362719.28-1502982.14) | 26176.81  (25002.61-27576.09) | 39.83  (30.84 to 49.43) |
| Region |  |  |  |  |  |
| Central Europe, eastern Europe, and central Asia | 10396049.82  (9123457.42-11705395.57) | 1725.1  (1511.65-1944.99) | 25054330.93  (22165594.26-27979835.12) | 4123.95  (3609.73-4660.88) | 139.06  (99.89 to 185.9) |
| Central Asia | 2305459.23  (1728874.46-2806618.73) | 2800.48  (2100.82-3410.35) | 3216799.83  (2470845.33-3924042.44) | 3776.15  (2919.53-4602.6) | 34.84  (-2.38 to 86.25) |
| Central Europe | 3253396.22  (2562155.15-3982822) | 1610.27  (1264.08-1966.31) | 6542804.15  (5181792.95-7826749.32) | 3303.48  (2633.43-3975.72) | 105.15  (52.28 to 176.38) |
| Eastern Europe | 4837194.37  (4124978.13-5622757.22) | 1504.12  (1276.65-1747.24) | 15294726.95  (13000598.46-17477008.12) | 4712.68  (3952.79-5450.71) | 213.32  (150.69 to 291.59) |
| High income | 18578431.62  (18017916.16-19071093.58) | 1048.79  (1013.01-1088.53) | 22476795.48  (21744679.48-23841839.64) | 1283.33  (1223.34-1397.8) | 22.36  (13.3 to 32.15) |
| Australasia | 16089.5  (15260.95-16953.48) | 31.52  (29.72-33.6) | 62054.46  (50526.22-72604.42) | 124.2  (101.4-145.6) | 293.98  (226.36 to 375.61) |
| High-income Asia Pacific | 82472.83  (69327.34-100956.61) | 20.86  (17.1-26.06) | 474020.65  (425864.24-537116.61) | 123.76  (107.61-149.67) | 493.32  (351.15 to 680.29) |
| High-income North America | 10158008.97  (9773753.79-10564962.95) | 1927.7  (1850.72-2006.67) | 11722647.58  (11320929.69-12387581.61) | 2221.69  (2130.83-2401.42) | 15.25  (7.13 to 23.99) |
| Southern Latin America | 1363544.26  (1318038.34-1415607.88) | 1638.38  (1582.13-1703.83) | 2318475.46  (2261502.61-2419986.64) | 2758.91  (2681.61-2899.79) | 68.39  (59.5 to 77.78) |
| Western Europe | 6958316.06  (6695214.39-7213232.54) | 828.36  (792.56-872.42) | 7899597.34  (7514631.1-8417682.4) | 975.62  (909.43-1084.93) | 17.78  (6.35 to 30.43) |
| Latin America and Caribbean | 20434779.32  (18680502.02-22283038.25) | 3325.58  (3042.02-3623.29) | 31525753.39  (29028137.79-34749691.33) | 5013.06  (4614.48-5529.78) | 50.74  (32.85 to 71.05) |
| Andean Latin America | 4010935.31  (3458123.34-4675913.18) | 6719.2  (5787.89-7811.69) | 4473448.24  (4175306.93-4763860.17) | 7337.99  (6860.88-7775.15) | 9.21  (-7.21 to 28.54) |
| Caribbean | 522083.14  (296610.13-834668.09) | 1009.65  (572.59-1616.92) | 1972117.48  (1448535.39-2698251.44) | 3710.61  (2719.87-5092.55) | 267.52  (100.09 to 575.05) |
| Central Latin America | 10783698.3  (9201036.84-12376896.82) | 4292.49  (3667.88-4923.69) | 12929787.28  (10964099.82-15209032.49) | 5046.91  (4286.58-5927.55) | 17.58  (-5.52 to 46.32) |
| Tropical Latin America | 5118062.56  (4683488.87-5616780.58) | 2040.21  (1865.42-2241.72) | 12150400.38  (11301148.34-13299376.13) | 4691.27  (4356.12-5159.43) | 129.94  (102.75 to 160.78) |
| North Africa and Middle East | 13208762.4  (11441393.57-14930754.19) | 2778.67  (2416.26-3127.27) | 19839053.12  (17553135.65-22515710.09) | 4015.87  (3561.3-4514.41) | 44.52  (21.38 to 72.08) |
| South Asia | 36221508.10  (33517287.78-39497924.17) | 2395.44  (2223.2-2588.22) | 58645299.12  (54457266.58-65885774.19) | 3778.09  (3536.35-4191.74) | 57.72  (40.52 to 77.02) |
| Southeast Asia, east Asia, and Oceania | 4194850.53  (1796733.36-8426161.54) | 160.39  (70.37-319.18) | 17429571.47  (12210192.93-26687741.48) | 663.79  (464.86-1005.95) | 313.86  (72.32 to 894.01) |
| East Asia | 621270.22  (147728.21-3142317.33) | 31.52  (7.45-157.37) | 49673.6  (12853.16-160386.7) | 2.84  (0.77-9.17) | -90.99  (-99.45 to 48.36) |
| Oceania | 35750.65  (10020.84-73587.55) | 421.36  (123.65-861.88) | 352531.87  (123111.98-679095.51) | 3979.92  (1415.16-7665.59) | 844.55  (191.27 to 2963) |
| Southeast Asia | 3537829.66  (1564295.71-6585581.43) | 524.11  (229.57-979.78) | 17027366  (11981704-26045951.3) | 2515.25  (1774.4-3850.19) | 379.91  (110.07 to 996.35) |
| Sub-Saharan Africa | 20318380.24  (18682571.16-22055972.88) | 3659.21  (3396.43-3922.18) | 37038792.46  (33744570.04-41926438.45) | 6398.04  (5899.95-6988.55) | 74.85  (56.42 to 95.44) |
| Central Sub-Saharan Africa | 2530986.71  (2256037.67-2839553.79) | 3891.28  (3486.08-4318.01) | 4283384.76  (3621539.91-5064033.81) | 6333.55  (5441.27-7395.46) | 62.76  (34.91 to 96.37) |
| Eastern Sub-Saharan Africa | 7228483.66  (6659401.86-7865048.23) | 3595.8  (3343.51-3882.69) | 15663398.98  (13891142.12-17661609.57) | 7476.07  (6688.62-8190.62) | 107.91  (83.42 to 135.68) |
| Southern Sub-Saharan Africa | 3489125.8  (3411095.17-3577596.79) | 5744.82  (5633.56-5867.11) | 6241677.85  (5806148.6-6678701.94) | 9952.56  (9312.19-10560.85) | 73.24  (62.19 to 85.05) |
| Western Sub-Saharan Africa | 7069784.06  (6374060.46-7963997.68) | 3039.44  (2752.64-3335.42) | 10850330.86  (9704599.05-12440234.73) | 4496.38  (4072.06-4973.47) | 47.93  (28.78 to 69.94) |
| **YLL** |  |  |  |  |  |
| Age group |  |  |  |  |  |
| Early Neonatal | 30.62  (28.69-33.18) | 1.23  (1.15-1.33) | 52.91  (49.08-57.5) | 2.16  (2-2.35) | 75.32  (57.36 to 95.34) |
| Late Neonatal | 282.25  (262.65-308.15) | 3.81  (3.55-4.16) | 483.39  (448.11-528.74) | 6.63  (6.14-7.25) | 73.81  (54.78 to 95.19) |
| 1-5 months | 15217.94  (14149.29-16474.6) | 27.83  (25.88-30.13) | 25725.37  (23781.88-28240.79) | 47.85  (44.24-52.53) | 71.93  (53.17 to 92.99) |
| 6-11 months | 20905.72  (19350.17-22866.79) | 32.39  (29.98-35.43) | 35204.36  (32451.61-38480.05) | 55.71  (51.35-60.89) | 72.00  (52.55 to 93.93) |
| 12-23 months | 88366.58  (82299.88-94702.76) | 67.23  (62.61-72.05) | 145625.54  (135355.64-157993.33) | 113.41  (105.41-123.04) | 68.68  (51.91 to 87.31) |
| 2-4 years | 736021.98  (690021.46-794967.74) | 179.85  (168.61-194.25) | 1245622.57  (1158400.06-1358175.38) | 309.04  (287.4-336.96) | 71.83  (54.35 to 91.29) |
| 5-9 years | 583843.44  (477129.13-671229.65) | 85.74  (70.07-98.58) | 943282.47  (724779.5-1123920.74) | 137.29  (105.49-163.59) | 60.12  (22.35 to 109.55) |
| 10-14 years | 662920.43  (533360.09-761695.53) | 100.36  (80.75-115.32) | 1116434.73  (827855.56-1304852.52) | 167.47  (124.18-195.74) | 66.87  (26.82 to 119.55) |
| 15-19 years | 1234995.85  (1174520.08-1314299.17) | 200.1  (190.3-212.95) | 1947165.66  (1836355.42-2069599.62) | 312.06  (294.3-331.68) | 55.95  (43.61 to 69.34) |
| 20-24 years | 1726517.02  (1645326.48-1828224.44) | 289.76  (276.14-306.83) | 2755659.69  (2600975.26-2928143.05) | 461.46  (435.56-490.35) | 59.26  (47.08 to 72.44) |
| 25-29 years | 3000429.74  (2853521.42-3190415.23) | 507.64  (482.79-539.79) | 4921515.41  (4657229.35-5239842.2) | 836.5  (791.58-890.61) | 64.78  (51.87 to 78.79) |
| 30-34 years | 4690717.06  (4456108.95-5012528.13) | 776.56  (737.72-829.84) | 7967196.41  (7557140.52-8502129.99) | 1318.02  (1250.19-1406.52) | 69.73  (56.07 to 84.57) |
| 35-39 years | 5924102.96  (5626959.63-6325699.84) | 1080.89  (1026.67-1154.16) | 10314346.58  (9794263.34-11020427.51) | 1839  (1746.27-1964.89) | 70.14  (56.47 to 85) |
| 40-44 years | 7913830.45  (7503185.74-8453055.1) | 1601.37  (1518.27-1710.48) | 13746308.04  (13045718.76-14744990.78) | 2747.88  (2607.84-2947.52) | 71.6  (57.43 to 87.03) |
| 45-49 years | 8941583.95  (8451357.02-9577653.6) | 1890.06  (1786.43-2024.51) | 15286639.55  (14488545.97-16442443.05) | 3228.4  (3059.85-3472.5) | 70.81  (56.15 to 86.84) |
| 50-54 years | 9687044.84  (9154453.48-10389611.36) | 2203.34  (2082.2-2363.14) | 16314650.81  (15433012.04-17572986.62) | 3666.85  (3468.69-3949.67) | 66.42  (51.88 to 82.36) |
| 55-59 years | 11267959.3  (10634786.68-12073845.06) | 2933.54  (2768.7-3143.35) | 18924270.43  (17902049.44-20400586.45) | 4782.14  (4523.83-5155.2) | 63.02  (48.71 to 78.7) |
| 60-64 years | 13315233.03  (12583287.25-14221108.91) | 4221.14  (3989.1-4508.32) | 22397925.01  (21199046.82-24095004.51) | 6998.31  (6623.71-7528.56) | 65.79  (51.64 to 81.27) |
| 65-69 years | 13731342.79  (13002072.19-14697612.5) | 5094.03  (4823.49-5452.5) | 23310812.11  (22128189.23-24993795.74) | 8450.78  (8022.05-9060.9) | 65.9  (52.05 to 81) |
| 70-74 years | 11727058.81  (11138019.34-12490708.81) | 5981.78  (5681.32-6371.31) | 19836412.20  (18887357.42-21106060.92) | 9636.85  (9175.78-10253.66) | 61.1  (48.67 to 74.58) |
| 75-79 years | 8618060.5  (8210904.74-9159855.93) | 6662.05  (6347.31-7080.88) | 13784990.01  (13133437.76-14640315.37) | 10452.33  (9958.29-11100.87) | 56.89  (45.18 to 69.55) |
| 80-84 years | 6885272.23  (6574937.15-7288133.77) | 7983.57  (7623.73-8450.69) | 11111028.25  (10617411.16-11741061.59) | 12686.28  (12122.68-13405.63) | 58.9  (47.81 to 70.83) |
| 85-89 years | 4235021.77  (4048676.37-4469100.48) | 9497.06  (9079.18-10021.98) | 6535943.53  (6246842.97-6882827.17) | 14295.03  (13662.73-15053.71) | 50.52  (40.41 to 61.35) |
| 90-94 years | 2417545.41  (2312660.99-2537609.74) | 13861.03  (13259.68-14549.42) | 3672240.26  (3518421.5-3863355.02) | 20527.49  (19667.66-21595.81) | 48.09  (38.62 to 58.22) |
| ≥95 years | 975557.07  (933016.59-1020302.94) | 18660.16  (17846.46-19516.05) | 1417200.5  (1354104.7-1493678.2) | 26002.21  (24844.55-27405.39) | 39.35  (30.38 to 48.93) |
| Region |  |  |  |  |  |
| Central Europe, eastern Europe, and central Asia | 10026376.29  (8785813.09-11278911.54) | 1646.41  (1443.51-1852.34) | 23621666.78  (20940804.33-26187993.93) | 3823.05  (3380.65-4251.04) | 132.2  (96.21 to 174.81) |
| Central Asia | 2213555.60  (1612191.29-2719249.44) | 2703.61  (1981.63-3312.78) | 2970855.45  (2211014.4-3581559.96) | 3518.91  (2626.46-4234.37) | 30.16  (-6.97 to 82.11) |
| Central Europe | 3180067.6  (2452499.33-3897738.52) | 1556.57  (1201.16-1911.23) | 6165426.94  (4811249.71-7478426.5) | 3026.7  (2370.46-3672.13) | 94.45  (42.12 to 166.03) |
| Eastern Europe | 4632753.1  (3907438.16-5463729.96) | 1419.75  (1192.58-1680.88) | 14485384.39  (12447135.51-16657982.13) | 4377.29  (3733.44-5069.81) | 208.31  (144.98 to 288.02) |
| High income | 18241692.43  (17797616.93-18658703.43) | 1020.68  (994.59-1045.18) | 21414019.67  (21083708.89-21777209.41) | 1195.19  (1176.89-1216.46) | 17.1  (13.66 to 20.64) |
| Australasia | 15674.3  (14866.59-16384.72) | 30.31  (28.69-31.72) | 60840.28  (49401.6-71224.77) | 120.72  (98.05-141.32) | 298.36  (230.72 to 379.83) |
| High-income Asia Pacific | 76286.72  (64366.05-93330.51) | 18.09  (15.09-22.29) | 431535.31  (394309.84-472897.46) | 104.84  (96.27-114.68) | 479.45  (366.11 to 620.35) |
| High-income North America | 10003061.85  (9656013.14-10358032.54) | 1889.16  (1822-1957.55) | 11213162.68  (10996038.8-11446529.72) | 2096.01  (2055.42-2140.17) | 10.95  (6.47 to 15.61) |
| Southern Latin America | 1340723.04  (1300116.33-1383730.33) | 1606.46  (1558.41-1657.83) | 2243600.12  (2219487.58-2267984.12) | 2655.12  (2626.32-2683.84) | 65.28  (59.95 to 70.79) |
| Western Europe | 6805946.52  (6573379.16-7020597.29) | 797.42  (769.34-822.1) | 7464881.28  (7257143.99-7687022.36) | 887.9  (862.7-915.78) | 11.35  (6.49 to 16.42) |
| Latin America and Caribbean | 19824513.11  (18115689.71-21558277.92) | 3226.08  (2948.6-3506.32) | 30091738.42  (27934571.09-32808885.04) | 4782.03  (4440.64-5212.66) | 48.23  (31.7 to 66.84) |
| Andean Latin America | 3907454.5  (3363342.26-4563294.57) | 6561.57  (5653.1-7651.92) | 4294413.09  (4049510.22-4504092.7) | 7069.78  (6668.9-7413.22) | 7.75  (-8.29 to 26.59) |
| Caribbean | 505168.72  (266166.26-823457.68) | 974.8  (512.28-1590.59) | 1926926.67  (1403455.06-2657438.48) | 3619.2  (2625.46-5007.6) | 271.27  (95.07 to 606.65) |
| Central Latin America | 10529868.93  (9014953.95-12126731.39) | 4194.38  (3598.12-4825.49) | 12320338.92  (10375891.11-14489070.21) | 4813.56  (4063.42-5650.6) | 14.76  (-7.94 to 43.06) |
| Tropical Latin America | 4882020.95  (4520231.14-5298081.21) | 1941.9  (1799.32-2106.93) | 11550059.74  (10799086.1-12291346.55) | 4444.82  (4154.83-4731.05) | 128.89  (106.62 to 153.56) |
| North Africa and Middle East | 12602045.65  (10824064.2-14091556.66) | 2676.58  (2301.95-2983.31) | 18546948.96  (16240537.1-20601873.43) | 3800.47  (3332.96-4216.74) | 41.99  (19.51 to 68.7) |
| South Asia | 34429382.17  (32217549.81-36621847.33) | 2297.85  (2149.8-2445.39) | 53538240.94  (51470619.88-56056975.25) | 3503.49  (3370.9-3666.39) | 52.47  (41.18 to 64.66) |
| Southeast Asia, east Asia, and Oceania | 4018723.69  (1633682.19-8253837.23) | 152.19  (62.24-311.55) | 16352342.94  (11407042.75-25670273.15) | 613.54  (429.3-958.87) | 303.13  (59.73 to 917.47) |
| East Asia | 606404.93  (136294.58-3126802.3) | 30.57  (6.77-156.39) | 46279.84  (9858.94-157721.6) | 2.63  (0.58-9.04) | -91.39  (-99.54 to 60.86) |
| Oceania | 35243.11  (9608.88-73139.5) | 417.26  (120.22-859.08) | 336784.84  (91064.42-654470.13) | 3862.9  (1065.8-7496.55) | 825.77  (174.63 to 3020.77) |
| Southeast Asia | 3377075.65  (1410230.28-6432856.83) | 501.52  (206.62-958.25) | 15969278.26  (11261426.37-25004450.52) | 2368.32  (1673.5-3704.99) | 372.23  (99.15 to 1019.75) |
| Sub-Saharan Africa | 19257128.41  (17953760.08-20678734.01) | 3545.39  (3310.35-3799.89) | 34191778.1  (31639812.1-36743805.78) | 6098.73  (5651.93-6543.13) | 72.02  (55.57 to 90.21) |
| Central Sub-Saharan Africa | 2365247.5  (2116610.94-2631190.21) | 3742.08  (3370.09-4158.86) | 3915498.01  (3398128.12-4614407.34) | 6009.07  (5217.25-7086.72) | 60.58  (33.07 to 93.77) |
| Eastern Sub-Saharan Africa | 6938551.51  (6429350.92-7520026.14) | 3512.63  (3258.76-3803.13) | 14451525.93  (12992782.19-15598320.08) | 7137.71  (6411.8-7697.43) | 103.2  (80.44 to 128.84) |
| Southern Sub-Saharan Africa | 3432245.49  (3368014.15-3490995.81) | 5671.66  (5573.2-5762.87) | 6009843.74  (5633173.18-6301499.1) | 9659.93  (9086.5-10101.11) | 70.32  (61.19 to 79.97) |
| Western Sub-Saharan Africa | 6521083.91  (5906224.95-7105357.23) | 2899.51  (2628.8-3157.29) | 9814910.42  (8865004.06-10587398.82) | 4236.21  (3822.93-4570.33) | 46.10  (28.7 to 65.86) |

* Percentage change is the comparison of age-standardized rates for 2020 and 2021.

DALY, Disability-Adjusted Life Year; YLL, Years of Life Lost; CI, confidence interval.
