# Supplementary material for: Trends and cross-country inequities by region, sex, age in the mortality, incidence, and disability-adjusted life years of COVID-19: Analysis from the Global Burden of Disease Study 2021
Source: PLoS Negl Trop Dis. 2025 Oct 27;19(10):e0013642. doi: 10.1371/journal.pntd.0013642 (PMC12558479; doi:10.1371/journal.pntd.0013642)
Supplement: S3 Table — * P-values are comparisons of inequity indexes for 2020 and 2021. DALY, Disability-Adjusted Life Year; YLL, Years of Life Lost; SDI, sociodemographic index; CI, confidence interval. (DOCX) [file pntd.0013642.s009.docx]

**S3 Table. Slope and concentration indexes of the incidence, death, DALYs, and YLLs of COVID-19 by sex, SDI and region.**

|  | **Slope index (95% CI)** | | | **Concentration index (95% CI)** | | |
| --- | --- | --- | --- | --- | --- | --- |
|  | **2020** | **2021** | ***P* *** | **2020** | **2021** | ***P* *** |
| **Incidence** |  |  |  |  |  |  |
| All included | -14,151(-20,372 to -7931) | -13,769(-22,239 to -5299) | 0.943 | -0.251(-0.299 to -0.204) | -0.229(-0.272 to -0.186) | 0.499 |
| Sex |  |  |  |  |  |  |
| Male | -14,114(-20,315 to -7912) | -13,677(-22,130 to -5225) | 0.935 | -0.254(-0.301 to -0.206) | -0.233(-0.276 to -0.190) | 0.521 |
| Female | -14,190(-20,431 to -7950) | -13,830(-22,353 to -5308) | 0.947 | -0.249(-0.296 to -0.201) | -0.225(-0.268 to -0.183) | 0.478 |
| SDI |  |  |  |  |  |  |
| Low SDI | -23,164(-38,087 to -8241) | -2110(-23,035 to 18,815) | 0.108 | -0.091(-0.171 to -0.010) | -0.021(-0.094 to 0.052) | 0.208 |
| Low-middle SDI | -3118(-16,479 to 10,243) | -2259(-19,526 to 15,007) | 0.939 | -0.039(-0.085 to 0.006) | -0.001(-0.035 to 0.033) | 0.185 |
| Middle SDI | 12,589(-2270 to 27,448) | 2836(-11,355 to 17,027) | 0.352 | 0.117(0.003 to 0.231) | 0.024(-0.04 to 0.087) | 0.160 |
| High-middle SDI | 2105(-25,340 to 29,549) | 14,293(-21,193 to 49,778) | 0.594 | 0.522(0.344 to 0.701) | 0.559(0.379 to 0.739) | 0.778 |
| High SDI | -6751(-15,795 to 2292) | -10,544(-22,979 to 1891) | 0.629 | -0.224(-0.322 to -0.127) | -0.163(-0.263 to -0.063) | 0.389 |
| Region |  |  |  |  |  |  |
| Central Europe, eastern Europe, and central Asia | -14,633(-27,487 to -1779) | -13,228(-25,037 to -1420) | 0.875 | -0.001(-0.054 to 0.052) | 0.026(-0.017 to 0.069) | 0.440 |
| Central Asia | 6739(-27,376 to 40,855) | 7574(-18,312 to 33,459) | 0.970 | -0.050(-0.187 to 0.088) | 0.043(-0.055 to 0.141) | 0.283 |
| Central Europe | -18,201(-34,238 to -2164) | -20,196(-38,009 to -2382) | 0.870 | -0.040(-0.120 to 0.039) | -0.049(-0.089 to -0.009) | 0.853 |
| Eastern Europe | -15,648(-34,768 to 3473) | -9250(-29,365 to 10,865) | 0.651 | 0.049(-0.011 to 0.108) | 0.024(-0.013 to 0.060) | 0.485 |
| High income | 2139(-4847 to 9124) | 65(-10,595 to 10,724) | 0.750 | -0.137(-0.243 to -0.031) | -0.070(-0.159 to 0.019) | 0.341 |
| High-income Asia Pacific | -894(-3667 to 1879) | -4881(-8339 to -1423) | 0.078 | -0.182(-0.231 to -0.134) | -0.075(-0.121 to -0.028) | 0.001 |
| High-income North America | 4479(-30,351 to 39,309) | -52,784(-88,247 to -17,320) | 0.024 | -0.068(-0.081 to -0.056) | -0.059(-0.070 to -0.048) | 0.286 |
| Southern Latin America | 7654(-10,872 to 26,180) | -8319(-15,311 to -1328) | 0.114 | -0.010(-0.219 to 0.199) | -0.036(-0.087 to 0.016) | 0.815 |
| Western Europe | -171(-9532 to 9190) | 4649(-4736 to 14,034) | 0.476 | -0.086(-0.150 to -0.022) | 0.024(-0.026 to 0.073) | 0.007 |
| Latin America and Caribbean | -19,460(-30,329 to -8590) | -18,230(-27,609 to -8851) | 0.867 | -0.035(-0.108 to 0.038) | -0.092(-0.135 to -0.049) | 0.183 |
| Andean Latin America | -26,411(-68,810 to 15,988) | -32,440(-37,829 to -27,051) | 0.782 | -0.066(-0.182 to 0.051) | -0.117(-0.134 to -0.099) | 0.394 |
| Caribbean | -16,456(-26,127 to -6786) | -11,375(-28,543 to 5794) | 0.613 | -0.271(-0.419 to -0.122) | -0.089(-0.175 to -0.003) | 0.038 |
| Central Latin America | -1688(-17,755 to 14,378) | -18,619(-38,603 to 1366) | 0.196 | 0.081(-0.004 to 0.166) | -0.044(-0.130 to 0.043) | 0.043 |
| North Africa and Middle East | -14,086(-32,418 to 4246) | -13(-23,800 to 23,773) | 0.358 | -0.112(-0.215 to -0.01) | -0.027(-0.137 to 0.082) | 0.265 |
| South Asia | 16,706(-42,160 to 75,573) | 11,779(-79,956 to 103,513) | 0.929 | -0.030(-0.060 to 0.000) | -0.014(-0.046 to 0.017) | 0.472 |
| Southeast Asia, east Asia, and Oceania | 516(-560 to 1592) | 10,471(-5408 to 26,349) | 0.220 | -0.587(-0.828 to -0.346) | -0.578(-0.760 to -0.396) | 0.954 |
| East Asia | -34(-225to 156) | 221(-468 to 911) | 0.484 | -0.002(-0.032 to 0.028) | 0.213(-1.110 to 1.535) | 0.750 |
| Oceania | 8638(-12,308 to 29,584) | 23,022(-110,589 to 156,634) | 0.835 | 0.137(-0.296 to 0.571) | -0.095(-0.169 to -0.021) | 0.300 |
| Southeast Asia | -902(-8746 to 6942) | 3202(-13,427 to 19,831) | 0.662 | 0.038(-0.203 to 0.279) | 0.045(-0.08 to 0.170) | 0.960 |
| Sub-Saharan Africa | -2411(-13,581 to 8760) | 9885(-4474 to 24,243) | 0.185 | 0.029(-0.031 to 0.089) | -0.028(-0.086 to 0.030) | 0.178 |
| Central Sub-Saharan Africa | -3505(-26,030 to 19,020) | 17,493(-9593 to 44,578) | 0.243 | -0.054(-0.108 to 0.000) | 0.098(0.040 to 0.156) | 0.000 |
| Eastern Sub-Saharan Africa | 10,361(-8277 to 28,999) | 4803(-28,441 to 38,048) | 0.775 | 0.048(-0.072 to 0.168) | -0.020(-0.103 to 0.062) | 0.361 |
| Southern Sub-Saharan Africa | 14,402(-39,276 to 68,079) | -44,650(-85,503 to -3797) | 0.086 | 0.061(-0.005 to 0.127) | -0.120(-0.150 to -0.090) | 0.000 |
| Western Sub-Saharan Africa | -7266(-23,890 to 9358) | -1797(-22,047 to 18,453) | 0.682 | 0.037(-0.033 to 0.107) | -0.017(-0.087 to 0.053) | 0.285 |
| **Death** |  |  |  |  |  |  |
| All included | -117(-147 to -87) | -175(-212 to -137) | 0.018 | -0.286(-0.344 to -0.228) | -0.286(-0.336 to -0.237) | 0.989 |
| Sex |  |  |  |  |  |  |
| Male | -156(-195 to -116) | -234(-286 to -181) | 0.020 | -0.287(-0.345 to -0.228) | -0.291(-0.341 to -0.241) | 0.915 |
| Female | -80(-101 to -60) | -121(-148 to -95) | 0.017 | -0.274(-0.333 to -0.215) | -0.273(-0.325 to -0.222) | 0.980 |
| SDI |  |  |  |  |  |  |
| Low SDI | -196(-301 to -91) | -116(-234 to 2) | 0.324 | -0.134(-0.225 to -0.043) | -0.052(-0.113 to 0.009) | 0.144 |
| Low-middle SDI | -10(-84 to 64) | 35(-153 to 223) | 0.663 | -0.050(-0.118 to 0.017) | -0.018(-0.091 to 0.055) | 0.520 |
| Middle SDI | 48(-23 to 120) | 10(-75 to 95) | 0.498 | 0.227(0.080 to 0.374) | 0.063(-0.022 to 0.149) | 0.059 |
| High-middle SDI | -21(-116 to 73) | 43(-62 to 147) | 0.373 | 0.359(0.162 to 0.557) | 0.534(0.356 to 0.713) | 0.198 |
| High SDI | -16(-40 to 8) | -45(-75 to -14) | 0.147 | -0.205(-0.316 to -0.094) | -0.183(-0.288 to -0.079) | 0.781 |
| Region |  |  |  |  |  |  |
| Central Europe, eastern Europe, and central Asia | -83(-129 to -37) | -46(-111 to 20) | 0.361 | -0.110(-0.200 to -0.019) | 0.032(-0.038 to 0.102) | 0.015 |
| Central Asia | 19.647(-111 to 150) | 80(-47 to 207) | 0.515 | 0.046(-0.118 to 0.211) | 0.142(-0.044 to 0.328) | 0.450 |
| Central Europe | -50(-99 to 0) | -106(-212 to 0) | 0.344 | -0.06(-0.141 to 0.022) | -0.110(-0.192 to -0.029) | 0.390 |
| Eastern Europe | -37(-101 to 28) | -31(-113 to 52) | 0.910 | 0.059(-0.050 to 0.168) | 0.063(0.010 to 0.116) | 0.946 |
| High income | -1(-25 to 22) | -10(-294 to 10) | 0.597 | -0.150(-0.276 to -0.023) | -0.090(-0.214 to 0.033) | 0.511 |
| High-income Asia Pacific | -2(-8 to 5) | -19(-60 to 22) | 0.420 | 0.143(-0.007 to 0.294) | 0.216(-0.155 to 0.587) | 0.723 |
| High-income North America | 39(-99 to 178) | -12(-124 to 100) | 0.572 | -0.046(-0.057 to -0.035) | -0.059(-0.067 to -0.050) | 0.076 |
| Southern Latin America | 92(-12 to 197) | 8(-60 to 77) | 0.186 | 0.066(-0.098 to 0.230) | -0.022(-0.088 to 0.044) | 0.330 |
| Western Europe | -13(-43 to 16) | -14(-37 to 9) | 0.949 | -0.128(-0.225 to -0.031) | -0.018(-0.099 to 0.063) | 0.088 |
| Latin America and Caribbean | -109(-181 to -37) | -112(-177 to -46) | 0.950 | 0.029(-0.085 to 0.142) | -0.043(-0.109 to 0.024) | 0.286 |
| Andean Latin America | -148(-575 to 279) | -155(-1066 to 756) | 0.989 | -0.043(-0.216 to 0.131) | -0.015(-0.347 to 0.318) | 0.883 |
| Caribbean | -117(-196 to -39) | -111(-255 to 32) | 0.944 | -0.490(-0.639 to -0.34) | -0.133(-0.265 to 0.000) | 0.000 |
| Central Latin America | -49(-158 to 61) | -134(-227 to -40) | 0.247 | 0.094(-0.022 to 0.210) | -0.010(-0.088 to 0.067) | 0.140 |
| North Africa and Middle East | -82(-147 to -17) | -133(-226 to -40) | 0.380 | -0.184(-0.297 to -0.072) | -0.134(-0.221 to -0.047) | 0.489 |
| South Asia | 34(-279 to 346) | 66(-291 to 423) | 0.894 | -0.093(-0.213 to 0.027) | -0.007(-0.083 to 0.069) | 0.236 |
| Southeast Asia, east Asia, and Oceania | 1(-10 to 12) | -17(-73 to 38) | 0.531 | -0.522(-0.763 to -0.282) | -0.596(-0.798 to -0.394) | 0.645 |
| East Asia | -2(-3 to -1) | 4(-0 to 9) | 0.011 | -0.023(-0.042 to -0.003) | 0.502(-0.496 to 1.500) | 0.303 |
| Oceania | -11(-62 to 40) | -266(-463 to -69) | 0.014 | -0.116(-0.193 to -0.040) | -0.116(-0.164 to -0.069) | 0.998 |
| Southeast Asia | -4(-25 to 17) | -1(-72 to 70) | 0.944 | 0.078(-0.196 to 0.351) | -0.024(-0.195 to 0.147) | 0.536 |
| Sub-Saharan Africa | -33(-120 to 54) | 57(-55 to 168) | 0.212 | -0.023(-0.095 to 0.049) | -0.017(-0.089 to 0.056) | 0.903 |
| Central Sub-Saharan Africa | -147(-331 to 37) | -74(-136 to -12) | 0.462 | -0.072(-0.199 to 0.055) | 0.006(-0.033 to 0.045) | 0.249 |
| Eastern Sub-Saharan Africa | -63(-227 to 101) | -61(-279 to 156) | 0.989 | -0.009(-0.157 to 0.139) | -0.020(-0.112 to 0.072) | 0.898 |
| Southern Sub-Saharan Africa | 13(-604 to 630) | -842(-1636 to -49) | 0.095 | 0.031(-0.041 to 0.104) | -0.159(-0.210 to -0.108) | 0.000 |
| Western Sub-Saharan Africa | -155(-261 to -49) | -129(-233 to -26) | 0.735 | -0.128(-0.189 to -0.068) | -0.070(-0.135 to -0.004) | 0.197 |
| **DALY** |  |  |  |  |  |  |
| All included | -2713(-3392 to -2034) | -4044(-4921 to -3168) | 0.019 | -0.280(-0.338 to -0.221) | -0.276(-0.325 to -0.228) | 0.930 |
| Sex |  |  |  |  |  |  |
| Male | -3618(-4519 to -2718) | -5401(-6601 to -4200) | 0.020 | -0.281(-0.34 to -0.222) | -0.282(-0.332 to -0.232) | 0.976 |
| Female | -1846(-2303 to -1389) | -2765(-3391 to -2140) | 0.020 | -0.274(-0.332 to -0.216) | -0.265(-0.315 to -0.216) | 0.823 |
| SDI |  |  |  |  |  |  |
| Low SDI | -4432(-6812 to -2052) | -2638(-5359 to 82) | 0.331 | -0.132(-0.221 to -0.042) | -0.051(-0.111 to 0.009) | 0.143 |
| Low-middle SDI | -238(-1946 to 1470) | 742(-3590 to 5075) | 0.680 | -0.050(-0.116 to 0.016) | -0.018(-0.088 to 0.052) | 0.507 |
| Middle SDI | 1070(-604 to 2743) | 283(-1648 to 2214) | 0.546 | 0.213(0.062 to 0.365) | 0.055(-0.029 to 0.138) | 0.071 |
| High-middle SDI | -530(-2592 to 1532) | 992(-1434 to 3419) | 0.349 | 0.358(0.160 to 0.556) | 0.540(0.361 to 0.718) | 0.182 |
| High SDI | -371(-918 to 177) | -1067(-1774 to -361) | 0.127 | -0.195(-0.316 to -0.074) | -0.176(-0.290 to -0.062) | 0.823 |
| Region |  |  |  |  |  |  |
| Central Europe, eastern Europe, and central Asia | -1961(-2974 to -948) | -1172(-2650 to 307) | 0.388 | -0.114(-0.202 to -0.025) | 0.028(-0.041 to 0.098) | 0.013 |
| Central Asia | -76(-3152 to 2999) | 1449(-1751 to 4649) | 0.500 | 0.009(-0.157 to 0.175) | 0.115(-0.074 to 0.304) | 0.410 |
| Central Europe | -1233(-2407 to -59) | -2654(-4865 to -443) | 0.266 | -0.071(-0.157 to 0.015) | -0.112(-0.190 to -0.035) | 0.483 |
| Eastern Europe | -908(-2385 to 568) | -725(-2687 to 1237) | 0.884 | 0.056(-0.050 to 0.162) | 0.062(0.011 to 0.112) | 0.925 |
| High income | -17(-553 to 518) | -203(-687 to 281) | 0.614 | -0.132(-0.275 to 0.010) | -0.085(-0.221 to 0.052) | 0.635 |
| High-income Asia Pacific | -56(-206 to 94) | -391(-1266 to 484) | 0.460 | 0.062(-0.095 to 0.218) | 0.182(-0.130 to 0.494) | 0.498 |
| High-income North America | 842(-3074 to 4757) | -253(-3821 to 3316) | 0.685 | -0.056(-0.068 to -0.044) | -0.065(-0.074 to -0.056) | 0.221 |
| Southern Latin America | 2060(-154 to 4274) | 246(-1027 to 1519) | 0.164 | 0.070(-0.088 to 0.229) | -0.014(-0.068 to 0.040) | 0.323 |
| Western Europe | -264(-914 to 387) | -327(-853 to 199) | 0.882 | -0.123(-0.219 to -0.028) | -0.022(-0.101 to 0.057) | 0.109 |
| Latin America and Caribbean | -2450(-4132 to -767) | -2739(-4200 to -1278) | 0.799 | 0.040(-0.074 to 0.155) | -0.040(-0.101 to 0.021) | 0.223 |
| Andean Latin America | -2700(-12,885 to 7484) | -3638(-23,987 to 16711) | 0.936 | -0.027(-0.204 to 0.150) | -0.018(-0.341 to 0.306) | 0.959 |
| Caribbean | -2542(-4311 to -774) | -2458(-5685 to 770) | 0.964 | -0.487(-0.638 to -0.336) | -0.129(-0.260 to 0.002) | 0.000 |
| Central Latin America | -947(-3707 to 1814) | -2720(-5174 to -266) | 0.347 | 0.116(-0.005 to 0.237) | 0.024(-0.061 to 0.108) | 0.221 |
| North Africa and Middle East | -1881(-3311 to -452) | -2876(-5013 to -739) | 0.448 | -0.192(-0.307 to -0.077) | -0.126(-0.211 to -0.041) | 0.367 |
| South Asia | 870(-6241 to 7981) | 1636(-6714 to 9986) | 0.891 | -0.088(-0.204 to 0.028) | -0.005(-0.075 to 0.064) | 0.231 |
| Southeast Asia, east Asia, and Oceania | 4(-36 to 43) | -370(-1679 to 939) | 0.576 | -0.534(-0.768 to -0.300) | -0.598(-0.798 to -0.397) | 0.683 |
| East Asia | -43(-66 to -21) | 75(-10 to 160) | 0.009 | -0.022(-0.042 to -0.002) | 0.495(-0.519 to 1.509) | 0.318 |
| Oceania | -114(-1553 to 1324) | -6077(-10,721 to -1432) | 0.016 | -0.104(-0.196 to -0.013) | -0.115(-0.163 to -0.067) | 0.841 |
| Southeast Asia | -87(-589 to 414) | -5(-1699 to 1689) | 0.927 | 0.067(-0.193 to 0.328) | -0.020(-0.190 to 0.149) | 0.578 |
| Sub-Saharan Africa | -699(-2675 to 1276) | 1437(-1118 to 3993) | 0.195 | -0.019(-0.089 to 0.052) | -0.015(-0.085 to 0.056) | 0.939 |
| Central Sub-Saharan Africa | -3390(-7576 to 795) | -1720(-3084 to -355) | 0.457 | -0.075(-0.197 to 0.048) | 0.005(-0.032 to 0.042) | 0.221 |
| Eastern Sub-Saharan Africa | -1344(-5094 to 2407) | -1263(-6288 to 3763) | 0.980 | -0.003(-0.152 to 0.145) | -0.017(-0.107 to 0.073) | 0.878 |
| Southern Sub-Saharan Africa | 328(-13,783 to 14,439) | -19,195(-37,833 to -558) | 0.102 | 0.034(-0.039 to 0.106) | -0.156(-0.208 to -0.103) | 0.000 |
| Western Sub-Saharan Africa | -3419(-5827 to -1010) | -2844(-5195 to -494) | 0.738 | -0.118(-0.176 to -0.059) | -0.063(-0.126 to -0.001) | 0.214 |
| **YLL** |  |  |  |  |  |  |
| All included | -2635(-3299 to -1972) | -3920(-4775 to -3065) | 0.020 | -0.279(-0.339 to -0.220) | -0.278(-0.329 to -0.228) | 0.981 |
| Sex |  |  |  |  |  |  |
| Male | -3558(-4448 to -2668) | -5317(-6500 to -4135) | 0.020 | -0.281(-0.340 to -0.221) | -0.283(-0.334 to -0.233) | 0.947 |
| Female | -1754(-2189 to -1319) | -2625(-3208 to -2041) | 0.019 | -0.273(-0.333 to -0.213) | -0.268(-0.320 to -0.216) | 0.904 |
| SDI |  |  |  |  |  |  |
| Low SDI | -4347(-6685 to -2008) | -2579(-5224 to 67) | 0.326 | -0.133(-0.224 to -0.043) | -0.052(-0.114 to 0.010) | 0.145 |
| Low-middle SDI | -220(-1900 to 1461) | 763(-3499 to 5025) | 0.674 | -0.050(-0.118 to 0.017) | -0.019(-0.092 to 0.055) | 0.531 |
| Middle SDI | 1040(-592 to 2672) | 240(-1722 to 2202) | 0.539 | 0.217(0.064 to 0.370) | 0.054(-0.031 to 0.140) | 0.068 |
| High-middle SDI | -520(-2529 to 1488) | 906(-1388 to 3200) | 0.359 | 0.354(0.155 to 0.554) | 0.538(0.359 to 0.718) | 0.179 |
| High SDI | -357(-885 to 171) | -1013(-1678 to -349) | 0.129 | -0.193(-0.316 to -0.071) | -0.175(-0.291 to -0.059) | 0.831 |
| Region |  |  |  |  |  |  |
| Central Europe, eastern Europe, and central Asia | -1871(-2840 to -902) | -1096(-2533 to 341) | 0.381 | -0.116(-0.207 to -0.024) | 0.028(-0.045 to 0.101) | 0.015 |
| Central Asia | -55(-3076 to 2967) | 1357(-1812 to 4525) | 0.527 | 0.013(-0.159 to 0.185) | 0.121(-0.078 to 0.320) | 0.420 |
| Central Europe | -1173(-2321 to -25) | -2501(-4642 to -359) | 0.284 | -0.070(-0.157 to 0.017) | -0.118(-0.201 to -0.036) | 0.431 |
| Eastern Europe | -841(-2292 to 610) | -654(-2543 to 1235) | 0.877 | 0.056(-0.054 to 0.165) | 0.064(0.011 to 0.116) | 0.896 |
| High income | -23(-542 to 495) | -214(-668 to 240) | 0.587 | -0.131(-0.275 to 0.013) | -0.083(-0.224 to 0.057) | 0.641 |
| High-income Asia Pacific | -49(-211 to 112) | -378(-1257 to 502) | 0.472 | 0.104(-0.078 to 0.286) | 0.241(-0.141 to 0.622) | 0.527 |
| High-income North America | 832(-2999 to 4664) | -274(-3618 to 3071) | 0.670 | -0.056(-0.067 to -0.044) | -0.065(-0.075 to -0.056) | 0.190 |
| Southern Latin America | 2027(-135 to 4190) | 284(-961 to 1528) | 0.171 | 0.071(-0.087 to 0.229) | -0.012(-0.068 to 0.043) | 0.327 |
| Western Europe | -265(-889 to 359) | -318(-815 to 179) | 0.896 | -0.123(-0.220 to -0.027) | -0.019(-0.104 to 0.065) | 0.111 |
| Latin America and Caribbean | -2385(-4033 to -737) | -2467(-4078 to -857) | 0.945 | 0.043(-0.073 to 0.159) | -0.039(-0.101 to 0.023) | 0.220 |
| Andean Latin America | -2621(-12675 to 7433) | -3441(-23661 to 16779) | 0.943 | -0.027(-0.205 to 0.152) | -0.015(-0.348 to 0.319) | 0.951 |
| Caribbean | -2491(-4242 to -741) | -2369(-5572 to 834) | 0.948 | -0.491(-0.646 to -0.336) | -0.130(-0.266 to 0.005) | 0.000 |
| Central Latin America | -929(-3648 to 1791) | -2649(-5026 to -273) | 0.350 | 0.117(-0.005 to 0.239) | 0.024(-0.062 to 0.110) | 0.223 |
| North Africa and Middle East | -1872(-3298 to -447) | -2864(-4934 to -795) | 0.439 | -0.194(-0.311 to -0.077) | -0.132(-0.219 to -0.045) | 0.404 |
| South Asia | 809(-6123 to 7741) | 1530(-6356 to 9416) | 0.893 | -0.090(-0.209 to 0.030) | -0.005(-0.081 to 0.070) | 0.240 |
| Southeast Asia, east Asia, and Oceania | 3(-32 to 38) | -389(-1652 to 874) | 0.543 | -0.533(-0.768 to -0.297) | -0.599(-0.801 to -0.396) | 0.677 |
| East Asia | -43(-64 to -22) | 73(-8 to 155) | 0.007 | -0.023(-0.042 to -0.003) | 0.535(-0.535 to 1.606) | 0.307 |
| Oceania | -139(-1525 to 1247) | -5989(-10496 to -1481) | 0.015 | -0.106(-0.196 to -0.017) | -0.115(-0.163 to -0.067) | 0.863 |
| Southeast Asia | -84(-568 to 399) | -22(-1646 to 1603) | 0.942 | 0.069(-0.193 to 0.332) | -0.025(-0.198 to 0.147) | 0.554 |
| Sub-Saharan Africa | -682(-2636 to 1271) | 1358(-1149 to 3864) | 0.208 | -0.020(-0.093 to 0.052) | -0.015(-0.088 to 0.058) | 0.919 |
| Central Sub-Saharan Africa | -3379(-7510 to 752) | -1741(-3104 to -378) | 0.460 | -0.074(-0.202 to 0.053) | 0.004(-0.035 to 0.042) | 0.249 |
| Eastern Sub-Saharan Africa | -1359(-5051 to 2333) | -1332(-6238 to 3575) | 0.993 | -0.004(-0.154 to 0.146) | -0.018(-0.111 to 0.075) | 0.879 |
| Southern Sub-Saharan Africa | 254(-13748 to 14257) | -18995(-37670 to -320) | 0.106 | 0.033(-0.04 to 0.106) | -0.158(-0.212 to -0.104) | 0.000 |
| Western Sub-Saharan Africa | -3411(-5770 to -1052) | -2838(-5142 to -534) | 0.734 | -0.126(-0.186 to -0.066) | -0.068(-0.133 to -0.003) | 0.200 |

***** P-values are comparisons of inequity indexes for 2020 and 2021.

DALY, Disability-Adjusted Life Year; YLL, Years of Life Lost; SDI, sociodemographic index; CI, confidence interval.
