# Supplementary material for: Trends and cross-country inequities by region, sex, age in the mortality, incidence, and disability-adjusted life years of COVID-19: Analysis from the Global Burden of Disease Study 2021
Source: PLoS Negl Trop Dis. 2025 Oct 27;19(10):e0013642. doi: 10.1371/journal.pntd.0013642 (PMC12558479; doi:10.1371/journal.pntd.0013642)
Supplement: Attachment — Submitted filename: Response to Reviewers.docx [file pntd.0013642.s011.docx]

**Responses to editor and reviewers’ comments**

**Additional Editor Comments:**

In addition to addressing the comments by the reviewers, please also address this following concerns:

**[RESPONSE]:** We sincerely appreciate the editor for giving us the opportunity to revise the manuscript.

- The justification for joinpoint analysis and including scatterplots with the breakpoints and fitted lines. It is unclear if a breakpoint at 2020 was used.

**[RESPONSE]:** We sincerely thank the editor for the careful review and insightful feedback. The joinpoint regression was used to identify significant changes in the trend of all-cause disease burden from 1990 to 2021, particularly in the context of the COVID-19 pandemic, which may have altered longstanding patterns. This analytical approach enables the precise identification of structural changes in trends and assesses the timing and magnitude of any inflection points with statistical rigor. To improve the visualization of trend shifts, we have added scatterplots with fitted lines and explicitly marked breakpoints in the revised manuscript (Fig. S1). Regarding the breakpoint, we chose 2019 rather than 2020. Although the pandemic was first identified in December 2019 and escalated globally in 2020, the burden attributable to COVID-19 in 2019 was negligible and accounts for only a small proportion of total cases and deaths during the pandemic period. Therefore, we defined 2019 as the baseline or pre-pandemic reference year, against which changes in disease burden in 2020 and 2021 can be directly assessed. Moreover, as shown in Figure S1, the burden of disease exhibited a steady decline during 1990–2019 (pre-COVID-19 period) but reversed sharply during 2019–2021. This distinct change in trend further confirms the selection of 2019 as a statistically and epidemiologically meaningful inflection point. To improve clarity, we have stated this selection in the revised manuscript as follows: “**Although the first case of COVID-19 was identified in December 2019, the pandemic started in 2020. The year 2019 was chosen as the predefined breakpoint because it marks the end of the pre-pandemic trend and allows for directly comparing the subsequent years with 2019 to assess the substantial changes in disease burden during the COVID-19 pandemic (2020-2021)**”. (Methods section, Page 11, Lines 204-208)

- There should be clarification on what is meant by uncertainty interval (unclear if it refers to the empirical quantiles of the distribution across countries)

**[RESPONSE]:** The 95% uncertainty intervals (UIs) were derived from empirical quantiles (e.g., the 2.5th and 97.5th ranked values) based on either 1000 or 500 draws from posterior or simulation-based distributions, depending on the specific metric. These intervals reflected statistical uncertainty at the sampling level in the modelling process, rather than cross-country variability. For example, all-cause mortality and years of life lost (YLLs) estimates were derived from 1000 draws, whereas incidence and disability-adjusted life years (DALYs) in the GBD 2021 were based on 500 draws (GBD 2021 Demographics Collaborators, 2024; GBD 2021 Diseases and Injuries Collaborators, 2024). Notably, for COVID-19 mortality, the estimation involved an ensemble modelling approach and counterfactual analysis, where 100 draws of excess mortality were repeated ten times to produce 1000 draws (GBD 2021 Demographics Collaborators, 2024; GBD 2021 Causes of Death Collaborators, 2024). The uncertainty was propagated at each step of the estimation process.

Similarly to confidence interval (CI), UI conveys the statistical uncertainty associated with the estimates. To improve clarity and consistency in terminology, we have uniformly adopted the term CI instead of UI throughout the manuscript, as it is more widely used and easier to understand. Furthermore, we have added a detailed explanation of the interval derived in the GBD 2021 as follows: “**We collected the number of cases and deaths and the rates (per 100,000 population) and their corresponding 95% confidence intervals (CIs) of COVID-19 by sex, age, region, and country from 2020 to 2021. According to the GBD 2021 algorithm, these intervals were calculated as the 25th and 97.5th percentiles of 1000 or 500 draw-level estimates for each parameter generated through posterior or simulation-based distributions [13, 15, 20]**”. (Methods section, Page 9, Lines 169-174)

**Ref:**

[1] GBD 2021 Demographics Collaborators. Global age-sex-specific mortality, life expectancy, and population estimates in 204 countries and territories and 811 subnational locations, 1950-2021, and the impact of the COVID-19 pandemic: a comprehensive demographic analysis for the Global Burden of Disease Study 2021. Lancet 2024; 403(10440):1989-2056.

[2] GBD 2021 Diseases and Injuries Collaborators. Global incidence, prevalence, years lived with disability (YLDs), disability-adjusted life-years (DALYs), and healthy life expectancy (HALE) for 371 diseases and injuries in 204 countries and territories and 811 subnational locations, 1990-2021: a systematic analysis for the Global Burden of Disease Study 2021. Lancet 2024; 403(10440):2133-2161.

[3] GBD 2021 Causes of Death Collaborators. Global burden of 288 causes of death and life expectancy decomposition in 204 countries and territories and 811 subnational locations, 1990-2021: a systematic analysis for the Global Burden of Disease Study 2021. Lancet 2024; 403(10440):2100-2132.

**Reviewer #1:**

This manuscript uses available data from the Global Burden of Disease Study (GBD) 21 to investigate cross-country inequalities in mortality, incidence, and DALYS of COVID-19. this builds on previous work by the GBD and extends it with a focus on inequalities. The manuscript is well written and structured, and provides a large amount of data to cover the research objective from many different angles.

I do have some issues I would like to address:

**[RESPONSE]:** We appreciate the thoughtful review and constructive feedback provided by the reviewer.

**R1.1.** The authors mention a total number of COVID-19 incidence cases of 2.28 billion, this seems extremely high, compared to the WHO COVID-19 dashboard which currently reports a total cumulative worldwide incidence of only 777 million. I would like to see an explanation of why these numbers are so widely different. In contrast, the total number of deaths reported here (7.89 million) is much more similar to that reported by the WHO (7.08 million).

**[RESPONSE]:** We sincerely thank the reviewer for highlighting the apparent discrepancy between the estimates in GBD 2021 and those reported by the WHO. It is important to clarify that 2.28 billion incidence cases and 7.89 million deaths of COVID-19 were estimated for the year 2021 only, whereas the 777 million confirmed cases and 7.08 million deaths reported by WHO were cumulative totals up to 2025. Despite covering a shorter period, the estimates in GBD 2021 were much higher. This discrepancy primarily arises from differences in data sources and definitions of COVID-19 cases. Specifically, the WHO estimates were only based on officially reported confirmed cases and deaths of SARS-CoV-2 infection submitted by its Member States (World Health Organization, 2025). A “confirmed case” is defined as an individual with a positive nucleic acid amplification test (NAAT), or someone who meets clinical or epidemiological criteria and tests positive on a SARS-CoV-2 antigen rapid diagnostic test (Ag-RDT). These statistics heavily depend on national testing strategies, case definitions, reporting practices, and surveillance capacities, which may substantially underestimate the actual number of infections, especially in resource-limited settings or when official reporting has been scaled down. In contrast, the estimates presented in our study are based on the GBD 2021 framework, which uses an integrated modelling approach to estimate the actual number of infections and deaths by synthesizing multiple data sources, including reported cases and deaths, excess mortality rate attributable to COVID-19, hospital admissions, and seroprevalence surveys. In regions where data are unavailable, statistical models incorporating numerous covariates are used to predict disease burden, thus providing a more comprehensive and globally comparable estimate. To improve clarity, we have added the corresponding explanations in the revised manuscript as follows: “**In addition, COVID-19 became the leading cause of DALYs globally in 2021, with a total of 2.28 billion incidence cases and 7.89 million deaths attributed to COVID-19 in 2021 [13]. These estimates differed markedly from those reported by the World Health Organization (WHO), which were based on confirmed cases reported by national surveillance systems. In contrast, our estimates were derived from the GBD 2021 framework that integrates multiple data sources (including reported cases and deaths, hospital admissions, and representative seroprevalence surveys) to infer total infections and deaths, capturing both direct and indirect pandemic-related impacts [19]**”. (Discussion section, Page 26, Line 380- Page 27, Line 388). And we have provided a detailed description about how to estimate cases and deaths of COVID-19 in the GBD 2021 (Methods section, Page 8, Line 138- Page 9, Line 167), as in response to R1.5.

**Ref:**

[1] World Health Organization, 2025. WHO COVID-19 dashboard. Available from: https://data.who.int/dashboards/covid19/cases?n=o. Accessed June 25, 2025.

**R1.2.** Throughout the article, it seems that the authors are using the terms inequalities, inequities, and disparities interchangeably. Inequity has a stronger interpretation of social justice, so I would urge the authors to study this terminology and apply it consistently.

**[RESPONSE]:** We fully agreed that the terms “inequalities”, “inequities”, and “disparities” should be applied with conceptual clarity and consistency, particularly in the context of health research where they carry distinct meanings. According to the WHO, health inequities are differences in health status or in the distribution of health resources between different population groups, arising from the social conditions in which people are born, grow, live, work, and age (World Health Organization, 2018). Health inequities are unfair and could be reduced by government policies. In addition, WHO defines health inequalities as the metric by which inequities can be assessed (World Health Organization, 2013). Meanwhile, health disparities are generally regarded as systematic differences in any measurable aspect of health outcomes due to the different distribution of social conditions across these populations (Lee et.al, 2020). Based on these distinctions and the focus of our study, we consistently used the term “**inequity**” throughout the revised manuscript.

**Ref:**

[1] World Health Organization, 2018. Health inequities and their causes. Available from: https://www.who.int/news-room/facts-in-pictures/detail/health-inequities-and-their-causes. Accessed June 25, 2025.

[2] World Health Organization, 2013. Health inequality monitoring. Available from: https://www.who.int/docs/default-source/gho-documents/health-equity/handbook-on-health-inequality-monitoring/handbook-on-health-inequality-monitoring.pdf.

Accessed June 25, 2025.

[3] Lee H, Kim D, Lee S, Fawcett J. The concepts of health inequality, disparities and equity in the era of population health. Appl Nurs Res 2020; 56:151367.

**R1.3.** Both in the introduction and discussion, the authors talk about post-2019, and particularly in terms of long-term trends in health outcomes, I think this is somewhat misleading. The paper presents almost 30 years of pre-pandemic data, and compares it with two years of data gathered DURING the pandemic, and no data gathered POST-pandemic. It is very important to provide more discussion on this topic than only one paragraph in the limitations. The patterns found here represent the impact of the pandemic, but it might well be that these are short-lived and patterns will return to 'normal' in 2023 and beyond.

**[RESPONSE]:** We sincerely thank the reviewer for this critical insight. We agree that describing our findings as indicative of “post-2019” trends could mislead readers by implying inclusion of true post-pandemic recovery data. Accordingly, we have revised the terminology throughout the manuscript to specify “**post-2019 (i.e., 2019-2021)**”. In addition, we also added more discussion on this topic in the Discussion: “**Despite the evident impact of the COVID-19 pandemic, the global age-standardized disease burden was projected to continue declining since 2022, albeit at a slower rate than during the three decades preceding the COVID-19 pandemic [24]**”. (Discussion section, Page 27, Line 394-397). In addition, we have also further addressed this limitation in Discussion section: “**Additionally, while this study examined the acute impacts of the pandemic based on almost three decades of pre-pandemic data as well as two years of data during the pandemic, no post-pandemic data were available due to data accessibility limitations. Future longitudinal analyses based on accumulated data are expected to investigate the long-term impact of COVID-19 during the post-pandemic period, which will be crucial for informing public health policies**”. (Discussion section, Page 35, Line 556-561)

**R1.4.** The authors mention the GBD in the introduction, but do not provide much detail about what has and has not already been studied using these data. I would like to see a more thorough discussion of this, to make it clearer what the added value of the current study is, compared to previous studies using GBD 2021 data.

**[RESPONSE]:** We thank the reviewer for this valuable suggestion. The GBD 2021 conducted a comprehensive assessment of age- and sex-specific disease burdens in 204 countries and territories during 1990-2021, and it is the first time that GBD included COVID-19 as a health outcome. Previous studies using the GBD 2021 data have all focused on the general descriptive analysis of COVID-19 disease burden or explored the influence of the COVID-19 pandemic on the burden of other diseases like malaria, stroke, and depressive disorders. However, there remains a lack of quantitative evidence regarding the impact of inequities on the burden of COVID-19. Thus, this study addressed this research gap by investigating cross-country inequities in COVID-19 incidence, mortality, DALYs, and YLLs, utilizing data from the GBD 2021. We have now enriched the Introduction section in the revised manuscript: “**The GBD 2021 estimated the age- and sex-specific COVID-19 burdens in 204 countries and territories during the first two years of the COVID-19 pandemic (2020-2021) [13]. Previous studies based on the GBD 2021 have mainly focused on the general descriptive analysis of COVID-19 disease burden [13, 15] or the influence of the COVID-19 pandemic on the burden of other diseases like malaria [16], stroke [17], and depressive disorders [18]. However, there remains a lack of quantitative evidence regarding the socioeconomic-related inequities in the burden of COVID-19**”**.** (Introduction section, Page 6, Line 110- Page 7, Line 117)

**R1.5.** Moreover, in the methods, I would like to read more about the methodology of the GBD, how do they obtain their data, and how do they try to ensure it is internationally comparable?

**[RESPONSE]:** We recognized that a brief overview of the GBD methodology would help readers understand the disease fully. Accordingly, we considered supplementing the Methods section as follows: “

**Data input and Modelling**

**The GBD framework integrates multiple data sources and applies standardized ensemble modelling strategies to estimate the global burden of COVID-19 [13, 19]. The detailed description of the disease burden assessment methodology for COVID-19 has been published previously [13, 19].**

**In brief, the estimation of COVID-19 incidence leveraged diverse sources of data inputs, including reported cases, hospital admissions, and reported deaths (primarily from national databases and the Johns Hopkins University COVID-19 dashboard), as well as representative seroprevalence surveys identified through systematic reviews, SeroTracker, and governmental platforms. These data were adjusted for known biases, such as reporting delays, underreporting of deaths, waning antibody sensitivity, vaccination coverage, and reinfections. Infection-detection ratios (IDRs), infection-hospitalization ratios (IHRs), and infection-fatality ratios (IFRs) were empirically derived. For locations without input data, predictive models using published covariates were developed to estimate daily infections.**

**The estimation of COVID-19-related mortality was based on weekly or monthly all-cause mortality data from vital registration and surveillance reports. The International Classification of Diseases, tenth revision (ICD-10) codes for COVID-19 were defined as U07-U07.2. To address incomplete mortality reporting across regions, GBD employed a two-stage modelling framework that enables robust and comparable estimates across countries. For regions with input data, excess mortality was calculated as the difference between observed mortality and expected mortality, which was estimated using statistical models by assuming the pandemic did not occur. For regions without sufficient input data, a Bayesian model averaging (Rover model) was used to predict excess mortality based on 11 covariates, including COVID-19 pandemic covariates (e.g., seroprevalence) and background population health metrics (e.g., Healthcare Access and Quality Index). Excess deaths were calculated by multiplying excess mortality rates by the person-year exposure for the period**”. The methodology and two references have been added in the revised manuscript for readers seeking further methodological details. (Methods section, Page 8, Line 138- Page 9, Line 167)

**R1.6.** The SDI is a key concept in the manuscript, so I would like to read at least who conceived it and see a reference to a more thorough description of its methodology.

**[RESPONSE]:** The sociodemographic index (SDI), first introduced by GBD 2015 (GBD 2015 Mortality and Causes of Death Collaborators, 2016) and further refined in GBD 2019 (GBD 2019 Demographics Collaborators, 2020), is a summary indicator designed to capture the background social and economic conditions of a given location. It comprises three rescaled components: lag-distributed income (LDI) per capita, mean education attainment among individuals aged 15 and older (EDU15+), and the total fertility rate among women under the age of 25 (TFU25), which serves as a proxy for young women’s social status (e.g., access to education and labor force participation) (GBD 2021 Demographics Collaborators, 2024). Each component is normalized to a scale from 0 to 1 based on empirical thresholds derived from its relationships with selected health outcomes. The SDI is then calculated as the geometric mean of these three rescaled components. We have added the description of the SDI to the Methods section as follows: “**The sociodemographic index (SDI) is a composite measure developed by the GBD collaborators to reflect the socioeconomic and demographic development of a country or region [13]. It is calculated as the geometric mean of three rescaled indicators: lag-distributed income per capita, mean educational attainment among individuals aged 15 and older, and total fertility rate among women under the age of 25. Each component is normalized to a scale from 0 to 1 using empirically derived bounds**”. In addition, we have cited relevant references to provide readers with more comprehensive methodological details regarding the SDI. (Methods section, Page 10, Lines 184-190)

**Ref:**

[1] GBD 2015 Mortality and Causes of Death Collaborators. Global, regional, and national life expectancy, all-cause mortality, and cause-specific mortality for 249 causes of death, 1980–2015: a systematic analysis for the Global Burden of Disease Study 2015. Lancet 2016; 388(10053):1459–1544.

[2] GBD 2019 Demographics Collaborators. Global age-sex-specific fertility, mortality, healthy life expectancy (HALE), and population estimates in 204 countries and territories, 1950–2019: a comprehensive demographic analysis for the Global Burden of Disease Study 2019. Lancet 2020; 396(10258):1160–203.

[3] GBD 2021 Demographics Collaborators. Global age-sex-specific mortality, life expectancy, and population estimates in 204 countries and territories and 811 subnational locations, 1950-2021, and the impact of the COVID-19 pandemic: a comprehensive demographic analysis for the Global Burden of Disease Study 2021. Lancet 2024; 403(10440):1989-2056.

**R1.7.** I think (some of) the data currently presented in Table 1, could be better visualized in a line graph. Now there are only 3 columns, for 1990, 2019, and 2021. Comparing these makes it difficult to conceptualize the large difference in time-span between these measurements. Using a line-graph would make visible very clearly the long-term changes, and the abrupt impact of the COVID-19 pandemic.

**[RESPONSE]:** We sincerely thank the reviewer for the constructive suggestion. Table 1 was originally intended to simultaneously present both the estimates of disease burden and the average annual percentage change (including the COVID-19-adjusted version) over the past 30 years. These complementary indicators were difficult to fully capture in a line graph focused solely on disease burden, thus we believed the tabular format remains essential for a comprehensive understanding. Nonetheless, we fully agreed that a line graph can more clearly illustrate the long-term trends in all-cause disease burden and the abrupt impact of COVID-19 than a static table limited to three reference years. To address this, we have added a line graph to illustrate the long-term trends in disease burden from 1990 to 2021, which has been included as Figure S1 in the appendix.

**R1.8.** In Figure 1, the colours used blend in too well to make it possible to distinguish the different regions. Either use more contrasting colours, or perhaps choose to remove the regional categories from this graph and present only overall data.

**[RESPONSE]:** As suggested, we have revised Figure 1 by using more contrasting colors to enhance visual separation among regions.

**R1.9.** In figure 2, it is not clear to me how useful it is to present the left column of total case numbers, in an international comparison as these are so strongly influenced by population size and characteristics. The right column of age-standardized rates is much more useful. Also, the figure legend is much too small to read.

**[RESPONSE]:** We agree that these raw counts are heavily influenced by population size and demographic structure and are unsuitable for international comparisons. Therefore, we have removed the left panel of the total case numbers and presented the age-standardized rates (ASRs) and the percentage change from 2020 to 2021, both of which provide a more meaningful and nuanced perspective for cross-country comparisons. In addition, we have increased the font size of the legend to improve readability, as suggested.

**R1.10.** Table 3, overall I think it is useful and interesting to use the SDI as indicators of socioeconomic differences between countries, and using the slope index and concentration index as measures of inequality within groups. However, I do not see the added value of using both at the same time, to study the level of inequality within the same SDI category.

**[RESPONSE]:** We thank the reviewer for acknowledging our use of the slope index and concentration index to quantify SDI-related health inequity. Although both metrics are used to quantify health inequity, they capture conceptually distinct dimensions and thus offer complementary insights (World Health Organization, 2013). Specifically, the slope index is an absolute inequality measure that reflects the predicted difference in health outcomes between the highest and lowest SDI countries. The slope index shares the same unit as the health indicator itself, making it intuitively interpretable and easy to understand. In contrast, the concentration index is a relative indicator of inequity, ranging from -1 to +1, with 0 indicating perfect equity. It reflects the extent to which a health outcome is concentrated across socio-economic groups and is especially valuable for comparisons across regions or periods. Both indicators are widely used in the literature on health inequity. Reporting the slope index and concentration index together enables a more comprehensive and nuanced interpretation, particularly in cases where absolute and relative inequities may diverge. To further emphasize the conceptual and statistical distinctions between the two metrics, we have revised the relevant statement as follows: “**The slope index and concentration index are commonly used to assess absolute and relative health inequity within the GBD framework. We used both indices to quantify SDI-related inequity of the COVID-19 burden across countries**”. (Methods section, Page 11, Lines 218-220)

**Ref:**

[1] World Health Organization, 2013. Health inequality monitoring. Available from: https://www.who.int/docs/default-source/gho-documents/health-equity/handbook-on-health-inequality-monitoring/handbook-on-health-inequality-monitoring.pdf.

Accessed June 25, 2025.

**R1.11.** In the discussion, the authors devote a long paragraph to disparities in vaccination rates. I do think this is an important issue, as possible contributor to the observed patterns, and an important source of international inequities. But this is not based on data that is presented in the current manuscript, therefore I feel it is somewhat speculative and perhaps a bit too much detail. It would be nice if the mentioned vaccination rates could be included in the manuscript in a table or figure, rather than only as mentioned in one sentence in the discussion.

**[RESPONSE]:** Thanks for your kind reminder. In the revised manuscript, we have added a figure in the supplementary file to illustrate the global inequities in COVID-19 vaccination rates (Fig. S6). We have also revised the corresponding discussion in the manuscript as follows: “**Notably, the pronounced inequities in vaccination rates contribute to the significant inequities in COVID-19 burden worldwide. As demonstrated in Fig. S6, remarkable absolute and relative inequities associated with SDI were detected in the vaccination rate of the COVID-19 vaccine. In 2021, the slope index of vaccinated individuals with at least one dose of COVID-19 vaccine was 56.88 (95% CI 48.46-65.29), indicating a 56.88% higher vaccination rate in countries with the highest SDI compared to those with the lowest SDI [32]. Low SDI countries generally did not have sufficient manufacturing capacities of COVID-19 vaccines [33], making them rely on international vaccine supplies through market purchases or donations. However, low-income countries generally face severe financial constraints [34], and vaccine nationalism also constrains the international supplies of vaccine donations [35]. High-income countries have prioritized vaccinating their domestic populations and stockpiling vaccines, rather than donating COVID-19 vaccines to curb the spread of COVID-19 in other regions, thereby exacerbating existing global health inequities [36]. Due to the absence of a coordinated global strategy, COVID-19 vaccinations in lower-income countries were delayed by several months and progressed at a slower pace, which, in turn, increases their vulnerability to COVID-19 and its variants [37]**”. (Discussion section, Page 29, Line 448- Page 30, Line 464)

**R1.12.** In the limitations section, the issue of variations in data quality and consistency to me is a key point for the merits of this manuscript, therefore I think it deserves a more detailed discussion. Have the GBD data been previously validated? Is there any indication of how serious these variations in quality are? The authors mention regions with underreporting, but I would like to see them interpret how this could have impacted their results and conclusions. Without underreporting, would the observed inequalities be smaller, or even larger?

**[RESPONSE]:** The GBD 2021 employed a range of methods to enhance data quality and mitigate the impact of reporting inconsistency bias. Specifically, the GBD 2021 framework synthesizes multiple data sources, including reported cases and deaths, excess mortality rate attributable to COVID-19, hospital admissions, and seroprevalence surveys, combined with an integrated modelling approach to generate regionally consistent estimates. For countries and regions with limited or missing data, predictive models incorporating multiple covariates are employed to estimate disease burden, thereby ensuring that regions with data-sparse settings are not excluded from the global assessment. Although these model-based estimates cannot fully replace high-quality observational data, they help to minimize the risk of underestimating disease burden in underreported regions.

Meanwhile, as mentioned by the reviewer, the GBD modelling approach has undergone out-of-sample predictive validity testing. Specifically, data from each location are iteratively excluded, and the model is re-estimated to predict outcomes for the excluded region. For COVID-19 mortality, this validation procedure yielded a mean relative error of 10.2% and a root mean squared error (RMSE) of 0.00066 for the excess mortality rate, indicating good predictive performance and low bias. Similar out-of-sample validation approaches have also been applied to other indicators such as incidence, DALYs, and YLLs.

Given that disease burden in underreported regions is estimated through model-based predictions, the influence of underreporting on health inequity estimates is inherently integrated into the model’s predictive accuracy, rather than constituting an independent source of bias. Consequently, it becomes challenging to ascertain whether health inequity has been underestimated or overestimated. Nevertheless, the prediction model used in GBD 2021 effectively reduces this potential bias. We have revised the corresponding discussion as appropriate: “**Variations in data quality and reporting consistency across countries may introduce potential biases. However, by synthesizing multiple data sources combined with an integrated modelling approach, GBD 2021 used predictive models incorporating multiple covariates to generate regionally consistent estimates for countries and regions with limited or missing data. These predictive models were validated and demonstrated good performance [13]. These model-based estimates cannot fully replace high-quality observational data, although they substantially minimize the risk of underestimating disease burden in underreported regions**”. (Discussion section, Page 34, Lines 541-548).

**R1.13.** Like mentioned before, the discussion of long-term effects of the COVID-pandemic and a potential return to previously observed trends deserved a more detailed discussion.

**[RESPONSE]:** After more than three decades of steady improvements in global life expectancy and declining age-standardized death rates, COVID-19 reversed longstanding progress and disrupted deaths in 2021. However, as noted by the reviewer, recent studies indicate that the global age-standardized disease burden is projected to continue declining since 2022, albeit at a slower rate compared to the three decades prior to the pandemic. We have added more discussion in the Discussion section: “**Despite the evident impact of the COVID-19 pandemic, the global age-standardized disease burden was projected to continue declining since 2022, albeit at a slower rate than during the three decades preceding the COVID-19 pandemic [24]**”. (Discussion section, Page 27, Line 394-397). In addition, future analyses are expected to explore the long-term impact of COVID-19 as more post-pandemic data become available, we also further address this in the limitation section: “**Additionally, while this study examined the acute impacts of the pandemic based on almost three decades of pre-pandemic data as well as two years of data during the pandemic, no post-pandemic data were available due to data accessibility limitations. Future longitudinal analyses based on accumulated data are expected to investigate the long-term impact of COVID-19 during the post-pandemic period, which will be crucial for informing public health policies**”. (Discussion section, Page 34, Line 556- Page 35, Line 561)

**Reviewer #2:**

Thanks for the opportunity to revise this manuscript. It provides a broad analysis of global COVID-19 burden and inequalities using data from the GBD study. While it uses robust methods, is well-written and responds to crucial public health questions, it suitability to PLOS NTD is questionable as COVID-19 is not neglected or tropical disease. Also, it does not affect marginalized populations as COVID-19 affects everyone. The COVID-19 pandemic, while it is an impactful topic, does not align with the scope completely. While health equity is a cross-cutting theme, the lack of direct relevance to NTD weakens its fit for PLOS NTD.

**[RESPONSE]:** We sincerely thank the reviewer for their thoughtful feedback and for recognizing the methodological robustness and public health relevance of our study. We also appreciate the reviewer’s concern regarding the alignment of our manuscript with the scope of PLOS neglected tropical disease (NTD). In response, we would like to clarify that although COVID-19 is not traditionally classified as an NTD, our study addresses an equally critical and overlapping dimension with NTDs, which may be the reason why some papers on COVID-19 have been published in PLOS NTD. For detailed clarification, please refer to the following Response R2.1.

Major comments:

**R2.1.** The study does not address NTD. The authors should reframe the discussion to emphasize the implications for NTD affected population.

**[RESPONSE]:** The COVID-19 pandemic has disproportionately affected marginalized populations, particularly those in low- and middle-income countries, which also face significant burdens from neglected tropical diseases. Our research aims to identify and quantify the health inequities exacerbated by the pandemic, with a specific emphasis on socioeconomic inequities in health outcomes. This inequity-centered approach resonates strongly with one of the central missions of PLOS NTDs: to draw attention to the health challenges faced by underserved and neglected communities. Furthermore, recent studies have highlighted that the pandemic has intensified pre-existing structural vulnerabilities—such as inadequate healthcare infrastructure, delayed access to medical treatment, and persistent social marginalization—that mirror the systemic challenges commonly observed in NTD-endemic regions. In this context, the pandemic has significantly amplified existing health inequities, a theme that is highly aligned with the editorial philosophy of PLOS NTDs. Thus, many COVID-19-related studies have been previously published in PLOS NTDs. We have reframed the discussion to emphasize the implications for the NTD-affected population in the revised manuscript: “**Additionally, as indicated in our study, the COVID-19 pandemic has disproportionately affected marginalized populations, particularly in the source-poor countries of subtropics and tropics, which also face significant burdens from neglected tropical diseases (NTDs). Our study highlights similar socioeconomic-related inequities in the burden of COVID-19 with NTDs, underscoring the necessity of enhancing healthcare systems to effectively manage and eliminate both COVID-19 pandemics and NTDs**”. (Discussion section, Page 33, Line 533-538)

**R2.2.** Using data from the GBD 2021 is a strength but also a limitation. There is underreporting in low-income countries and could underestimate true inequalities. Although it is acknowledged by the author, it would be useful to discuss how such biases might skew inequality indices. If possible, include a sensitivity analysis or discussion about this potential limitation.

**[RESPONSE]:** We apologize for some confusion caused. Although underreporting of disease burden existed in low-income countries, the GBD 2021 employed a range of methods to enhance data quality and mitigate the impact of reporting inconsistency bias. Specifically, the GBD 2021 framework synthesizes multiple data sources, including reported cases and deaths, excess mortality rate attributable to COVID-19, hospital admissions, and seroprevalence surveys, combined with an integrated modelling approach to generate regionally consistent estimates. For countries and regions with limited or missing data, predictive models incorporating multiple covariates are employed to estimate disease burden, thereby ensuring that regions with data-sparse settings are not excluded from the global assessment. Although these model-based estimates cannot fully replace high-quality observational data, they help to minimize the risk of underestimating disease burden in underreported regions.

Meanwhile, the GBD modelling approach has undergone out-of-sample predictive validity testing. Specifically, data from each location are iteratively excluded, and the model is re-estimated to predict outcomes for the excluded region. For COVID-19 mortality, this validation procedure yielded a mean relative error of 10.2% and a root mean squared error (RMSE) of 0.00066 for the excess mortality rate, indicating good predictive performance and low bias. Similar out-of-sample validation approaches have also been applied to other indicators such as incidence, DALYs, and YLLs.

Since the disease burden in underreported regions is estimated through model-based predictions, the influence of underreporting on health inequity estimates is inherently integrated into the model’s predictive accuracy, rather than constituting an independent source of bias. Consequently, it becomes challenging to ascertain whether health inequity has been underestimated or overestimated. Nevertheless, the prediction model used in GBD 2021 effectively reduces this potential bias. We have revised the corresponding discussion as appropriate. (Discussion section, Page 34, Lines 542-548)

**R2.3.** The manuscript does not disentangle the components of the SDI. Stratify SDI components in supplementary analyses or discuss their relative contributions to COVID-19 outcomes.

**[RESPONSE]:** Thanks for your advice. In the revised manuscript, we have described the structure of the SDI and further explored health inequities associated with the three components of the SDI for a more detailed assessment, including mean educational attainment for those aged 15 and older (EDU15+), total fertility rate under the age of 25 (TFU25), and lag-distributed income (LDI). The results were presented in Fig S4. We observed very similar patterns of inequality associated with these three components. (Results section, Page 22, Lines 317-319)

**R2.4.** Justify why 2019 was chosen as the inflection point if the pandemic began in 2020.

**[RESPONSE]:** The year 2019 was a statistically and epidemiologically meaningful inflection point. A detailed justification was provided in the response to the editor.

Minor comments:

**R2.5.** Is there a difference between DALY and DLAY? I understand that the whole acronym is Disability-Adjusted Life Year.

**[RESPONSE]:** We are sorry for the typo about “DALY”. We have carefully reviewed the entire manuscript and corrected all the typos.

**R2.6.** Clarify where YLD estimates include long COVID-19 or only acute-phase disability.

**[RESPONSE]:** The YLD estimates include both acute-phase and post-acute (e.g., long-term) disability associated with COVID-19 (GBD 2021 Diseases and Injuries Collaborators, 2024). To enhance clarity, we have revised the relevant sentence in the manuscript as follows: “**The composite measure of DALYs was calculated by summing YLLs and years lived with disability (YLDs), which include both acute and long-term disability resulting from COVID-19**”. (Methods section, Page 10, Lines 180-182)

**Ref:**

[1] GBD 2021 Diseases and Injuries Collaborators. Global incidence, prevalence, years lived with disability (YLDs), disability-adjusted life-years (DALYs), and healthy life expectancy (HALE) for 371 diseases and injuries in 204 countries and territories and 811 subnational locations, 1990-2021: a systematic analysis for the Global Burden of Disease Study 2021. Lancet 2024; 403(10440):2133-2161.

**R2.7.** The conclusion mention “targeted interventions” but this statement lacks specificity. It would be useful to include some examples of equity-focused policies.

**[RESPONSE]:** Thanks for your suggestion. We have proposed more specific targeted interventions in the revised manuscript: “**For example: (1) Vaccine allocation strategies should prioritize regions with low SDI and large elderly populations, and incorporate mobile vaccination units to enhance access in underserved areas; (2) Community-based testing and early treatment programs in urban slums and rural regions, leveraging rapid antigen tests and antiviral drugs administered by local health workers, can effectively reach marginalized populations; (3) Protective policies targeting vulnerable groups, such as paid sick leave and workplace safety regulations in high-risk occupations (e.g., transportation and sanitation), where older men are disproportionately represented, can help reduce mortality inequities associated with occupational exposure**”. (Discussion section, Page 33, Lines 520-529)

**Reviewer #3:**

**[RESPONSE]:** We sincerely thank the thoughtful review and constructive feedback provided by the reviewer.

**R3.1.** Methods - When did you pull the GBD 2021 data? It was updated after the initial release. Please include the date.

**[RESPONSE]:** Thanks for your kind reminder. In the revised manuscript, we have added the data extraction date (July 13, 2024) to enhance the transparency and reproducibility of the study. To further ensure the robustness of our findings, we re-extracted the same data again on July 30, 2025, and confirmed that the estimates for COVID-19 and all-cause burden used in our analysis remained unchanged. This consistency indicates that our results are stable and unaffected by any subsequent database updates. (Methods section, Page 10, Line 197)

**R3.2.** Page 7 - line 121 - was long term COVID-19 recognized before the end of 2021?

**[RESPONSE]:** Yes, long-term COVID-19, also referred to as post-COVID-19 condition or long COVID, was indeed considered and incorporated into the GBD 2021 as part of the COVID-19 (Level 3). Although the clinical definition of long-term COVID-19 was formally published in April 2022 (Soriano et.al, 2022), it had already been adopted by the GBD research team in their internal estimation processes for the 2021 burden assessment (GBD 2021 Diseases and Injuries Collaborators, 2024). According to this definition, long-term COVID-19 occurs in individuals with a history of probable or confirmed SARS-CoV-2 infection, typically 3 months from the onset, with symptoms lasting at least 2 months and cannot be explained by an alternative diagnosis. Common symptoms include, but are not limited to, fatigue, shortness of breath, and cognitive dysfunction, which typically have an impact on everyday functioning.

**Ref:**

[1] Soriano JB, Murthy S, Marshall JC, Relan P, Diaz JV. A clinical case definition of post-COVID-19 condition by a Delphi consensus. Lancet Infect Dis 2022; 22(4):e102–e107.

[2] GBD 2021 Diseases and Injuries Collaborators. Global incidence, prevalence, years lived with disability (YLDs), disability-adjusted life-years (DALYs), and healthy life expectancy (HALE) for 371 diseases and injuries in 204 countries and territories and 811 subnational locations, 1990-2021: a systematic analysis for the Global Burden of Disease Study 2021. Lancet 2024; 403(10440):2133-2161.

**R3.3.** Page 9 lines 171 - 173 - These are widely used with GBD data but not particularly outside of it. Please provide evidence. Most studies don’t examine inequality on a linear scale (though they probably should). On line 174 is the rank the quintile?

**[RESPONSE]:** Thank you for your thoughtful comments. We agree that the slope index and concentration index are widely used within the GBD framework to assess health inequities, and their usage in studies based on other data sources exists but is relatively less common. To avoid overstating their general use, we have revised the relevant sentence in the manuscript as follows: “**The slope index and concentration index are commonly used to assess absolute and relative health inequity within the GBD framework**”. (Methods section, Page 11, Lines 218-219)

Regarding the scale of inequity assessment, the slope index is itself a linear measure of absolute inequity, while the concentration index is based on the nonlinear scale of the concentration curve. In most global health inequity studies, both indices are presented simultaneously to capture complementary dimensions of inequity—absolute and relative, respectively. Following this common practice, we adopted both indices to provide a more comprehensive understanding of inequity in the COVID-19 burden.

As for the “rank” mentioned in line 174, it is not a quintile but rather a relative position scale, defined as the midpoint of the range of each country’s cumulative population after sorting countries by SDI. This clarification has now been explicitly added in the revised manuscript as follows: “**The slope index of inequity was determined by linearly regressing country-level ASR due to COVID-19 on the proportion of the cumulative population after sorting all countries by SDI [21]**”. (Methods section, Page 11, Lines 220-222)

**R3.4.** The analysis did not include interactions between sex, age, etc. If the authors have the capability to include this in the adjusted analysis (which is not actually emphasized anywhere in the results so it isn’t clear if these results are adjusted or unadjusted for all variables), this would make the statement on page 28 lines 353-355 more accurate. Considering lines 445-452 (pages 32 and 33) specifically set up the idea that narrowing down exactly which populations need help, looking at the interactions would help policymakers know that it isn’t just males that need help but what particular subgroups need help. Is it just that males have high rates or is it something like older males need help but younger females need help? Because of the difference in the outcomes by sex, it would be very informative to discuss how the overall results (example incidence by SDI quintile) are also different by sex or why looking at that in the future would help inform policy.

**[RESPONSE]:** We acknowledge the importance of analyzing interaction effects to identify more specific high-risk subpopulations. However, our study was based on publicly available country-level summary data stratified by sex, age, SDI, and region from GBD 2021. The GBD dataset does not provide a multi-dimensional covariate structure that would allow for modelling complex interaction effects (i.e., sex × age or sex × age × SDI). Consequently, our approach is descriptive in nature and interactions could not be directly modelled in the current analysis. We have clarified in the revised manuscript that reported disparities are based on stratified estimates by age or sex and should be interpreted as marginal effects rather than interaction effects. We have also carefully revised relevant statements in the Discussion section to avoid any potential misinterpretation. In the limitation section, we have explicitly mentioned that future research incorporating multivariable and interaction-based modelling methods would be valuable for identifying more nuanced risk subpopulations, such as heightened vulnerability among older males or younger females. (Discussion section, Page 34, Lines 552-556)

**R3.5.** With so much information in the tables, it is a bit difficult to follow what I’m looking at and trace back what is written to the right place in the table. This is especially true for Table 3. Is there a way to focus the tables even more on what’s the most impactful? In Table 3, what is the p-value from?

**[RESPONSE]:** To improve the focus and readability of the tables, we have transformed part of the tabular information into visual formats: (1) for Table 1, we added a line chart showing age-standardized rates across the three years to better illustrate trend changes (see Figure S1); and (2) for Table 3, we have transformed it into a bar chart to visually present the regional variation, which helps to more effectively communicate the spatial patterns of health inequity (see Figure 4). The p-values presented in Table 3 were calculated to assess the statistical significance of the differences in health inequity indexes (e.g., slope index and concentration index) between 2020 and 2021.

**R3.6.** Page 10 line 196 - what do you mean “regions with different SDI levels”?

**[RESPONSE]:** In the original sentence, the “regions with different SDI levels” referred to five groups of countries categorized by quintiles of the sociodemographic index (SDI): Low SDI, Low-middle SDI, Middle SDI, High-middle SDI, and High SDI regions. These SDI-based regional classifications are commonly used in GBD analyses to reflect varying levels of socioeconomic development. To improve clarity, we have revised the manuscript as follows: “**Before COVID-19, the AAPC in mortality rates was relatively balanced across five SDI-based regions**”. (Results section, Page 13, Lines 246-247)

**R3.7.** Page 12 lines 206-207 - How is premature death something that contributes to disease burden? Isn’t it the other way around?

**[RESPONSE]:** We are sorry for this confusion. In the GBD framework, DALYs were calculated as the sum of YLLs due to premature death and years lived with disability (YLDs). In our analysis, YLLs accounted for 93.27% of total DALYs, indicating that premature death was the predominant component of the overall disease burden (i.e., DALYs) attributable to COVID-19. To better reflect this structural contribution and avoid suggesting a causal relationship, we have revised the sentence in the manuscript as follows: “**YLLs accounted for 93.27% of total DALYs, indicating that premature death was the predominant component of the disease burden attributable to COVID-19**”. (Results section, Page 15, Lines 258-260)

**R3.8.** Page 12 line 208 - Are the “four indicators” the incidence, etc? This isn’t clear. Similarly, on page 18 lines 264, is this true for the “four metrics”?

**[RESPONSE]:** The “four indicators” and “four metrics” refer to the same set of disease burden measures: incidence, deaths, DALYs, and YLLs. These four metrics were consistently applied as the main indicators in our evaluation of the COVID-19 burden and associated inequities. To avoid ambiguity, we have revised the relevant sentences by explicitly listing the four indicators at their first mention.

**R3.9.** Page 18 line 265-267 - Inequity would happen by sex not within each sex (unless you specifically are looking at stratifying each sex by something such as ethnicity) so saying “inequity was greater for males than females” isn’t correct.

**[RESPONSE]:** In this analysis, we stratified the population by sex and evaluated SDI-related inequity within each sex group using the slope index and concentration index. The results indicated that the degree of within-sex socioeconomic inequality in the COVID-19 burden was generally larger for males than for females. To avoid conceptual confusion, we have revised the sentence to: “**Among sex-stratified analyses, males exhibited greater SDI-related inequity in COVID-19 burden compared to females (e.g., DALYs: -5401 vs. -2765 for slope index; -0.282 vs. -0.265 for concentration index), except absolute inequity for incidence**”. (Results section, Page 22, Lines 319-322)

**R3.10.** Despite the emphasis on examining the global differences (not necessarily disparities) of the impact of COVID-19 by social, economic, and demographic groups, the method used includes very little of this information. The authors do use the SDI but this measure barely scratches the surface of either of these three categories (social, economic, or demographic). Understandably measuring some of these items and having comparable categories or items across such varied countries and regions is difficult if not impossible. However, it is very important for the authors to make this a major discussion point rather than limiting it to a single sentence (page 32 lines 437-439). To help with this, make more definitive statements in prior paragraphs in the discussion section that cover within and between country differences in socioeconomic determinants and try to better tie these paragraphs together. On the other hand, be very careful to not overstate the impact of the findings or make major discussion points that are not backed up by the evidence from the work of the authors. On lines 457-458 (pages 33) there is a “maybe” that the SDI could oversimplify the issue - IHME states on their website that it definitely does. Health issues such as hypertension and substance use were not included in the analysis, but are highlighted as very important to the outcomes in the authors’ analyses and areas for future intervention. The authors highlight smoking and drinking several times though these variables were not included in the study but are very vague about other health issues that were major contributors to poor COVID-19 outcomes (and that are also highly impacted by socioeconomic and sociocultural determinants). There are mentions of cardiovascular health but not other conditions that impact respiratory health such as COPD or asthma.

**[RESPONSE]:** Thanks for the insightful review. Firstly, we agreed with the reviewer that SDI oversimplifies complex socio-economic contexts. Thus, we have removed the “may” in the corresponding sentence. In addition, we further explored health inequities associated with the three components of the SDI for a more detailed assessment, including mean educational attainment for those aged 15 and older (EDU15+), total fertility rate under the age of 25 (TFU25), and lag-distributed income (LDI). The results were presented in Fig. S4. We observed very similar patterns of inequality associated with these three components (Results section, Page 22, Lines 317-319). Secondly, this study primarily focuses on cross-country differences rather than within-country variations. We have carefully reorganized the discussion section as suggested. Thirdly, we have thoroughly revised the manuscript to avoid overstating the implications of our findings. Fourthly, we have included examples of health issues, behavioral patterns, and lifestyle factors to illustrate the potential mechanisms underlying our results. For instance, sex differences in COVID-19 mortality rates may be attributed to variations in behavioral and lifestyle factors, such as smoking and alcohol consumption, or the prevalence of comorbidities, such as hypertension and cardiovascular diseases. However, due to limitations in data availability, we are unable to verify these potential mechanisms. Nevertheless, these explanations have been supported by previous studies. To prevent misinterpretation, we have carefully revised the relevant sections and cited corresponding references. (Discussion section, Page 28, Lines 428-429; Page 31, Lines 473-476)

**R3.11.** On page 27, lines 326-328, the authors state there is “compelling evidence” but do not bring back up what that evidence is.

**[RESPONSE]:** Sorry for the confusion caused. As indicated by GBD 2021, the global age-standardized disease burden has consistently shown a declining trend during 1990-2019 but experienced a remarkable shift in this trend during 2019-2021. However, the AAPCs during the COVID-19 pandemic became insignificant after excluding the cases and deaths of COVID-19 (Table 1). In addition, COVID-19 became the leading cause of DALYs globally in 2021, with a total of 2.28 billion incidence cases and 7.89 million deaths attributed to this disease. This represents a substantial disease burden that significantly reversed the previous global trend. In addition, our results also indicated that the increasing trend of mortality during the COVID-19 pandemic was not significant after excluding the cases and deaths of COVID-19 (Table 1). We have expanded the discussion and provided corresponding evidence in the manuscript. (Discussion section, Page 26, Lines 373-380)

**R3.12.** Page 27, lines 337-338 - This sentence is not clear. What do you mean?

**[RESPONSE]:** We have revised the sentence as **“While the age-standardized COVID-19 incidence rate demonstrated a decreasing trend with advancing age among the elderly (over 65 years), the corresponding age-standardized mortality rate increased rapidly with age”**. (Discussion section, Page 27, Lines 399-401)

**R3.13.** Page 28 lines 342-344 - Were the elderly more aware? This analysis uses data from 2020 and 2021 - is there a difference across time given the newness of the situation and the general confusion in 2020 of what to do? Nobody was sure of what to do or who was being more cautious especially across countries.

**[RESPONSE]:** Indeed, there is no evidence to show who was more aware. To prevent potential confusion, we have omitted this statement.

**R3.14.** Page 28 lines 349-351 - What differences in immune responses?

**[RESPONSE]:** The higher rate of mortality in men with COVID-19 may partly be explained by sex differences in immune responses. Previous studies indicated that there are sex differences in the detection of nucleic acids by innate immunity, with pattern recognition receptors (PRRs) playing a role [1]. The Toll-like receptor 7 (TLR7) gene may escape X inactivation, resulting in higher expression levels of TLR7 in females than in males. Moreover, in vitro experiments have shown that the exposure of peripheral blood mononuclear cells (PBMCs) to TLR7 ligands causes higher production of interferon-α (IFN-α) in cells in women than in men, and plasmacytoid dendritic cells (pDCs) from female humans have higher basal levels of IFN regulatory factor 5 (IRF5) and IFN-α production following TLR7 ligand stimulation. A review that examined the association between sex differences in the immune system concluded that sex-based immunological differences contribute to variations in susceptibility to infectious diseases. In the revised manuscript, we have provided this possible explanation and cited two relevant papers. **“The potential reasons may involve sex differences in immune responses (e.g., higher expression levels of the Toll-like receptor 7 in females) and behavioral and lifestyle factors [26, 27]”**. (Discussion section, Page 28, Line 410-412)

**R3.15.** On page 29, lines 378-381 - Expanding this though would really help make the emphasis on disparities stronger in this discussion.

**[RESPONSE]:** Thank you for your suggestion. We have extensively expanded the discussion of the potential reasons for health inequities in the burden of COVID-19. We have also carefully reorganized the sentences to better integrate these discussions. (Discussion section, Page 28, Line 428 - Page 28, Line 446)

**R3.16.** Page 30 line 384 - What is meant by resource-limited? In this US for example, hospitals were overwhelmed whether or not they were normally resource limited. What kind of resources do you mean?

**[RESPONSE]:** Thank you for highlighting this ambiguity. We define "resource-limited" as settings characterized by long-term structural deficiencies in healthcare infrastructure and workforce capacity, as evidenced by consistently low baseline service availability relative to population health needs. This distinction should be made from temporary capacity strain observed in well-resourced healthcare systems, such as US hospitals expanding ICU beds during periods of high demand. To avoid ambiguity, we revised the sentence as “**Lastly, but importantly, the lower resilience of healthcare systems in low SDI countries to manage surges in demand during pandemics, characterized by long-term structural deficiencies in healthcare infrastructure and workforce capacity, is a key determinant of cross-country inequities in the burden of COVID-19**”. (Discussion section, Page 29, Lines 443-446)

**R3.17.** Page 30 lines 384-386 - Please quantify and better explain the relationship with non-remote work. It’s obvious to me as someone well versed in disparities and equity, but potentially not clear to other readers.

**[RESPONSE]:** As suggested, we have revised the sentence as “**For example, people in low SDI countries are more likely to engage in non-remote work and therefore benefit less from lockdown restrictions compared to those who are able to work at home, which results in unequal exposure risk of COVID-19 [6, 29]**”. (Discussion section, Page 29, Lines 434-437)

**R3.18.** Page 30 lines 390-393 - Vaccination rates were not included in this study. This paragraph is definitely important for the discussion about why disparities exist for COVID-19 but seems out of place. Please tie it in better. For lines 394-396 - how did this exacerbate disparities? Which disparities? Line 397 - for all vaccination campaigns or just COVID-19? How does this differ from “normal”?

**[RESPONSE]:** Thanks for your valuable suggestion. In the revised manuscript, we have conducted an additional analysis to explore the inequity in vaccination rates of the COVID-19 vaccine. The results were presented in Fig. S6. We have also carefully revised the corresponding discussion in the manuscript: “**Notably, the pronounced inequities in vaccination rates contribute to the significant inequities in COVID-19 burden worldwide. As demonstrated in Fig. S6, remarkable absolute and relative inequities associated with SDI were detected in the vaccination rate of the COVID-19 vaccine. In 2021, the slope index of vaccinated individuals with at least one dose of COVID-19 vaccine was 56.88 (95% CI 48.46-65.29), indicating a 56.88% higher vaccination rate in countries with the highest SDI compared to those with the lowest SDI [32]. Low SDI countries generally did not have sufficient manufacturing capacities of COVID-19 vaccines [33], making them rely on international vaccine supplies through market purchases or donations. However, low-income countries generally face severe financial constraints [34], and vaccine nationalism also constrains the international supplies of vaccine donations [35]. High-income countries have prioritized vaccinating their domestic populations and stockpiling vaccines, rather than donating COVID-19 vaccines to curb the spread of COVID-19 in other regions, thereby exacerbating existing global health inequities [36]. Due to the absence of a coordinated global strategy, COVID-19 vaccinations in lower-income countries were delayed by several months and progressed at a slower pace, which, in turn, increases their vulnerability to COVID-19 and its variants [37]**”. (Discussion section, Page 29, Line 448 - Page 30, Line 464)

**R3.19.** Is the problem “vaccine nationalism”, a lack of a coordinated strategy for vaccine distribution, or distrust of the vaccine by different populations? How does these interact?

**[RESPONSE]:** Vaccine nationalism refers to the absence of a coordinated global strategy for vaccine distribution. Low SDI countries generally did not have sufficient manufacturing capacities of the COVID-19 vaccine and must rely on international vaccine supplies, such as donations. However, high-income countries have prioritized vaccinating their domestic populations and stockpiling vaccines (i.e., vaccine nationalism), rather than donating COVID-19 vaccines to curb the spread of COVID-19 in other regions, thereby exacerbating COVID-19 inequities. Due to the absence of a globally coordinated strategy, vaccination in lower-income countries started several months later and progressed at a slower pace, which, in turn, increases their vulnerability to COVID-19 and its variants. We have also carefully revised the corresponding discussion in the manuscript: “**Low SDI countries generally did not have sufficient manufacturing capacities of COVID-19 vaccines [33], making them rely on international vaccine supplies through market purchases or donations. However, low-income countries generally face severe financial constraints [34], and vaccine nationalism also constrains the international supplies of vaccine donations [35]. High-income countries have prioritized vaccinating their domestic populations and stockpiling vaccines, rather than donating COVID-19 vaccines to curb the spread of COVID-19 in other regions, thereby exacerbating existing global health inequities [36]”**. (Discussion section, Page 30, Lines 454-461)

**R3.20.** Page 31 lines 407-408 - Is the “frequent” a raw number or a rate? Provide that here. Is there more than one manuscript to cite that points to smoking and cardiovascular disease? Why are these important for the point you’re making?

**[RESPONSE]:** The “frequent” refers to the smoking rate. These points were used to explore the potential reasons for the greater inequities in COVID-19 mortality rates among males than females. Males experienced disproportionately higher mortality rates of COVID-19 than females due to various reasons, and males are more likely to suffer from severe COVID-19-associated outcomes, such as deaths and ICU admissions. Thus, the male population requires more medical resources, such as ICU equipment, to combat the threat of COVID-19. However, these resources are clearly insufficient in low-SDI countries, thereby exposing males to greater health inequities of COVID-19. We have revised the manuscript to clarify the discussion. (Discussion section, Page 30, Line 472 - Page 31, Line 481)

**R3.21.** Page 31 line 409 - Remove the word “may”. This is a true statement.

**[RESPONSE]:** As suggested, we have removed the word “may”.

**R3.22.** Page 31 starting with line 421 - Find a better way to discuss absolute and relative differences in the discussion. This section should provide more clarity to what you mean.

**[RESPONSE]:** Thanks for your suggestion. We have revised the discussion as suggested. (Discussion section, Page 32, Lines 496-502)

**R3.23.** Page 32 lines 441-447 - I expected the example starting on line 445 to be more specific about what this study showed in terms of the impact of these factors. Don’t be vague here.

**[RESPONSE]:** We have specified the key findings of this study and proposed more specific targeted interventions accordingly. (Discussion section, Page 33, Lines 520-529)

**R3.24.** Page 33 lines 468-470 - Burden of what?

**[RESPONSE]:** The “burden” specifically refers to the health burden attributable to COVID-19, and we have clarified the description in the revised manuscript.

**R3.25.** There are a few sentences and statements in the introduction and discussion that seem to be the work of others and should be cited as such. Also the methods section related to GBD is mostly from the GBD website and should be cited. They also describe their set up in other papers so you could just say this is described elsewhere.

**[RESPONSE]:** As suggested, we carefully reviewed the introduction, methods, and discussion sections and have added appropriate references where statements were informed by the GBD website or previous GBD-related publications, e.g., in Lines 95, 140, 483.

**R3.26.** This paper should be reviewed and edited for English. There are quite a few misspellings and awkward sentences.

**[RESPONSE]:** We have carefully reviewed and revised the entire manuscript to correct misspellings, improve grammar, and enhance sentence clarity and readability.

**R3.27.** In the abstract, please define SDI.

**[RESPONSE]:** As suggested, we have added the definition of SDI to the abstract as follows: “**The SDI is a composite development indicator that encompasses income, educational attainment, and fertility conditions**”. (Abstract section, Page 2, Lines 30-31)

**R3.28.** Throughout the abstract, paper, graphs and tables - please spell DALYs correctly.

**[RESPONSE]:** We have thoroughly reviewed the entire manuscript and corrected all typos.

**R3.29.** The confidence interval and uncertainty interval are the same thing - please be consistent in the use of one or the other. Except for early on, you mostly use the CI.

https://statmodeling.stat.columbia.edu/2022/04/05/confidence-intervals-compatability-intervals-uncertainty-intervals/

**[RESPONSE]:** In the original manuscript, we used the terms “confidence interval (CI)” and “uncertainty interval (UI)” in different sections to reflect the statistical uncertainty of the estimates: UI for metrics from GBD 2021 and CI for regression-derived results. To ensure consistency of terminology, we used the more widely-used term “confidence interval (CI)” instead of UI throughout the revised manuscript.
