# Supplementary material for: Trends and cross-country inequities by region, sex, age in the mortality, incidence, and disability-adjusted life years of COVID-19: Analysis from the Global Burden of Disease Study 2021
Source: PLoS Negl Trop Dis. 2025 Oct 27;19(10):e0013642. doi: 10.1371/journal.pntd.0013642 (PMC12558479; doi:10.1371/journal.pntd.0013642)
Supplement: Attachment — Submitted filename: Response_to_Reviewers_auresp_2.docx [file pntd.0013642.s012.docx]

**Responses to editor and reviewers’ comments**

**Reviewer #1:**

I commend the authors on a very thorough revision, I highly appreciate the very detailed and reference-backed responses to the issues raised by me and the other reviewer. I think the quality of the manuscript has improved substantially. I have no further issues, except that in reading the edited sections, I noticed that in the methods section:

**[RESPONSE]:** We sincerely thank the reviewer for the very positive feedback. We are delighted to know that you found the quality of the revised manuscript to have improved substantially and that no further concerns were raised. We also truly appreciate the time and effort you have dedicated to reviewing our work throughout the revision process.

**R1.1.** change :Page 9, Lines 169-174, the authors mention calculating a 95% confidence interval, by using the 25th and 97.5th percentiles. I assume this is just a typo and they mean the 2.5th and 97.5th.

**[RESPONSE]:** We are sorry for the typo. We have corrected it as 2.5th and 97.5th percentiles in the revised manuscript.

**Reviewer #2:**

The authors have adequately addressed my concerns, and the manuscript is acceptable for publication, provided that the English is edited.

**[RESPONSE]:** We thank the reviewer for the positive evaluation of our revised manuscript and for recognizing that the substantive concerns have been adequately addressed. Regarding the English language editing, we have carefully conducted a comprehensive revision of the entire manuscript. Please refer to the following Response R2.1.

**R2.1.** please do another careful review of language and grammar.

**[RESPONSE]:** We have conducted a thorough line-by-line revision of the entire manuscript to refine grammar, improve sentence structure, and enhance overall clarity and readability.

**Reviewer #4:**

Excellent manuscript that provides a wealth of data on global disparities in health care relative to the COVID pandemic. The manuscript is very well written and presented and the figures are superior.

**[RESPONSE]:** Thank you for your positive feedback. We appreciate the thoughtful review and constructive feedback provided by the reviewer.

**R4.1.** change : Line 55-56: Is this overall cause or infectious disease specific disease burden?

**[RESPONSE]:** We are sorry for the confusion caused. In this study, we focused on only two indicators: all-cause disease burden and COVID-19-specific disease burden. The term “**overall disease burden**” in lines 55-56 referred to all-cause disease burden, highlighting that it declined significantly before 2019 but was subsequently reversed after 2019, underscoring the substantial impact of the COVID-19 pandemic. To avoid any potential misunderstanding, we have revised the manuscript and consistently replaced “**overall disease burden**” with “**all-cause disease burden**”. (Author summary section, Page 5, Line 63)

**R4.2.** Line 126: The GBD 2021 conducted -> The GBD 2021 provided

**[RESPONSE]:** As suggested, we have replaced “**conducted**” with “**provided**”.

**R4.3.** Line 564: delete “In conclusion” since that is the title of this section :)

**[RESPONSE]:** Thanks for your kind reminder. We have removed the word “**In conclusion**” as suggested to improve conciseness.
